# Exploring the structural basis to develop efficient multi-epitope vaccines displaying interaction with HLA and TAP and TLR3 molecules to prevent NIPAH infection, a global threat to human health

**DOI:** 10.1371/journal.pone.0282580

**Published:** 2023-03-15

**Authors:** Sukrit Srivastava, Sonia Verma, Mohit Kamthania, Ajay Kumar Saxena, Kailash C. Pandey, Veena Pande, Michael Kolbe

**Affiliations:** 1 Infection Biology Group, Indian Foundation for Fundamental Research Trust, RaeBareli, India; 2 Department for Structural Infection Biology, Centre for Structural Systems Biology (CSSB) & Helmholtz-Centre for Infection Research, Hamburg, Germany; 3 Protein Biochemistry & Engineering Lab, Parasite-Host Biology Group, ICMR-National Institute of Malaria Research, New Delhi, India; 4 Molecular Medicine Lab., School of Life Science, Jawaharlal Nehru University, New Delhi, India; 5 Kumaun University, Bheemtal, Nainital, Uttarakhand, India; 6 Faculty of Mathematics, Informatics and Natural Sciences, University of Hamburg, Hamburg, Germany; Italian National Research Council, ITALY

## Abstract

Nipah virus (NiV) is an emerging zoonotic virus that caused several serious outbreaks in the south asian region with high mortality rates ranging from 40 to 90% since 2001. NiV infection causes lethal encephalitis and respiratory disease with the symptom of endothelial cell-cell fusion. No specific and effective vaccine has yet been reported against NiV. To address the urgent need for a specific and effective vaccine against NiV infection, in the present study, we have designed two Multi-Epitope Vaccines (MEVs) composed of 33 Cytotoxic T lymphocyte (CTL) epitopes and 38 Helper T lymphocyte (HTL) epitopes. Out of those CTL and HTL combined 71 epitopes, 61 novel epitopes targeting nine different NiV proteins were not used before for vaccine design. Codon optimization for the cDNA of both the designed MEVs might ensure high expression potential in the human cell line as stable proteins. Both MEVs carry potential B cell linear epitope overlapping regions, B cell discontinuous epitopes as well as IFN-γ inducing epitopes. Additional criteria such as sequence consensus amongst CTL, HTL and B Cell epitopes was implemented for the design of final constructs constituting MEVs. Hence, the designed MEVs carry the potential to elicit cell-mediated as well as humoral immune response. Selected overlapping CTL and HTL epitopes were validated for their stable molecular interactions with HLA class I and II alleles and in case of CTL epitopes with human Transporter Associated with antigen Processing (TAP) cavity. The structure based epitope cross validation for interaction with TAP cavity was used as another criteria choosing final epitopes for NiV MEVs. Finally, human Beta-defensin 2 and Beta-defensin 3 were used as adjuvants to enhance the immune response of both the MEVs. Molecular dynamics simulation studies of MEVs-TLR3 ectodomain (Human Toll-Like Receptor 3) complex indicated the stable molecular interaction. We conclude that the MEVs designed and *in silico* validated here could be highly potential vaccine candidates to combat NiV infections, with great effectiveness, high specificity and large human population coverage worldwide.

## Introduction

Nipah virus (NiV) is an emerging zoonotic virus of the genus Henipavirus of the Paramyxoviridae family [1]. NiV infection causes fatal encephalitis and respiratory disease with a particular symptom of endothelial cell-cell fusion [2]. The first NiV infection to human was reported in Malaysia in 1998. Later NiV outbreak was reported from Meherpur, Bangladesh in 2001. In the Malaysia NiV infection, the transmission was primarily due human contact with pigs, whereas in later outbreaks of Bangladesh and India the transmission was associated with contaminated date palm sap and human-to-human contact [3]. Bats are identified as the main reservoir for the NiV and being responsible for the transmission of the infection to both humans and animals [4]. After 2001 NiV outbreaks have been reported from different district of Bangladesh almost every year (2003–05, 2007–12). Till March 31, 2012, a total of 209 confirmed cases of NiV infections were reported out of which 161 people died resulting in the mortality rate as high as 77%. After several threats in Bangladesh, in total three NiV outbreaks have also been reported from India. Two of them occurred in the state of West Bengal in 2001 and 2007 [5]. The most recent NiV outbreak was reported from the Kerala state of India during the period of May to June-2018. The Kerala outbreak claimed 17 lives leaving only two survivors out of 19 confirmed cases [6]. Till present, there has no specific vaccine reported against NiV infection, and the pathogenesis mechanism of NiV to human cells is largely unknown. Hence, an immune-informatics approach investigating the potential of different NiV proteins for vaccine design would be an important and essential step forward for vaccine development.

NiV infection of human cells involves several protein-protein interactions and protein cluster formation on the host cell surface. Essential proteins involved in NiV pathogenesis include C protein, Fusion glycoproteins (F), Glycoproteins (G), Matrix proteins (M), Nucleocapsid protein, Phosphoprotein, Polymerase, V protein and the W protein [7–21]. The C protein regulates the early host pro-inflammatory response as well as the pathogen virulence thus providing a conducive environment for a successful NiV infection [7]. The attachment glycoprotein (G), the fusion protein (F) and the matrix protein (M) together form a cluster on the human cell membrane facilitating virus particle assembly and pathogenesis [8–12]. The G and F proteins of NiV have been shown to be immunogenic by inducing protective immune responses in hamsters [22,23]. The NiV matrix protein is observed to play a central role in virus particle formation and is essentially required for viral budding from the infected human cells [13–15]. The NiV Polymerase is responsible for the initiation of RNA synthesis, primer extension, and transition to elongation mode and hence the enzyme facilitates viral pathogenesis and survival in host cells [16]. The phosphoprotein and the glycoprotein of NiV are crucially involved in the regulation of viral replication [17,18] while the V protein of NiV is responsible for the host interferon (IFN) signaling evasion during pathogenesis [19,20]. Interestingly, the identical N-terminal region of the pathogen’s V and W protein is sufficient to exert the IFN-antagonist activity [21]. Hence, all the nine above mentioned NiV proteins are crucial in different ways for viral pathogenesis and might be important drug and vaccine candidates.

The Nipah virus is a zoonotic RNA virus and it infects human respiratory epithelium cells as well as differentiated neurons (in the brain and spinal cord). Thus, as concluded from previous animal model studies, the recovery from viral infection and the clearance of viral RNA requires the presence of virus-specific antibodies and interferon gamma (IFN-γ) secretion from T cells [24–26]. Along with the B cell, the T cell also play a critical role in immune response against NiV infection. In recent studies a number of B cell and T cell epitopes from the NIPAH proteome have been reported [27–38]. Further different approaches were proposed for the design of multi epitope vaccines [39–44]. However, the proposed vaccines utilized a very limited number (6 to 8) of T and B cell epitopes targeting any one NiV protein. The use of limited number of epitopes could be challenging for the successful presentation of the exogenous vaccine candidates in view of the proteolytic cleavage by Antigen Presenting Cells (APC).

In the present study we have screened for the most potential Cytotoxic T lymphocyte (CTL) epitopes, Helper T lymphocyte (HTL) and B cell epitopes from the NiV proteome. We have shortlisted and prioritized the most potential and highest scoring 33 CTL, 38 HTL and 16 B cell epitopes. Out of these a total of novel 61 CTL and HTL epitopes, targeting nine NiV proteins were used to design CTL and HTL Multi-Epitope Vaccines (MEVs). Both the MEVs carry potential B cell linear epitope overlapping regions, B cell discontinuous epitopes as well as IFN-γ inducing epitopes. Here, the additional criteria of consensus of epitope amongst CTL, HTL and B Cell epitopes to chose final epitopes was also implemented. The designed MEVs carry potential to elicit cell-mediated as well as humoral immune response. We further evaluated several critical properties including the IC(50), immunogenicity, conservancy, Non-toxic to identify the epitopes with the highest immunogeneic potential against NiV. The chosen overlapping epitopes were validated by analyzing molecular interaction with HLA allele binders and with human Transporter Associated with Antigen Processing (TAP). These criteria for choosing and validating the CTL epitopes for NiV MEVs is novel and was not reported hitherto. The shortlisted epitopes were utilized for the design of CTL and HTL Multi-Epitope Vaccines against NiV. The designed vaccines were further studied for their stable interaction with innate immunity Human Toll-Like Receptor 3 (TLR3). The analysis of cDNA of the designed Multi-Epitope Vaccine has predicted to be highly favorable for expression in mammalian cell line. Overall in the present study we have designed and proposed potential Multi-Epitope Vaccines against Nipah virus infection.

## Materials and methods

**Fig 1:**
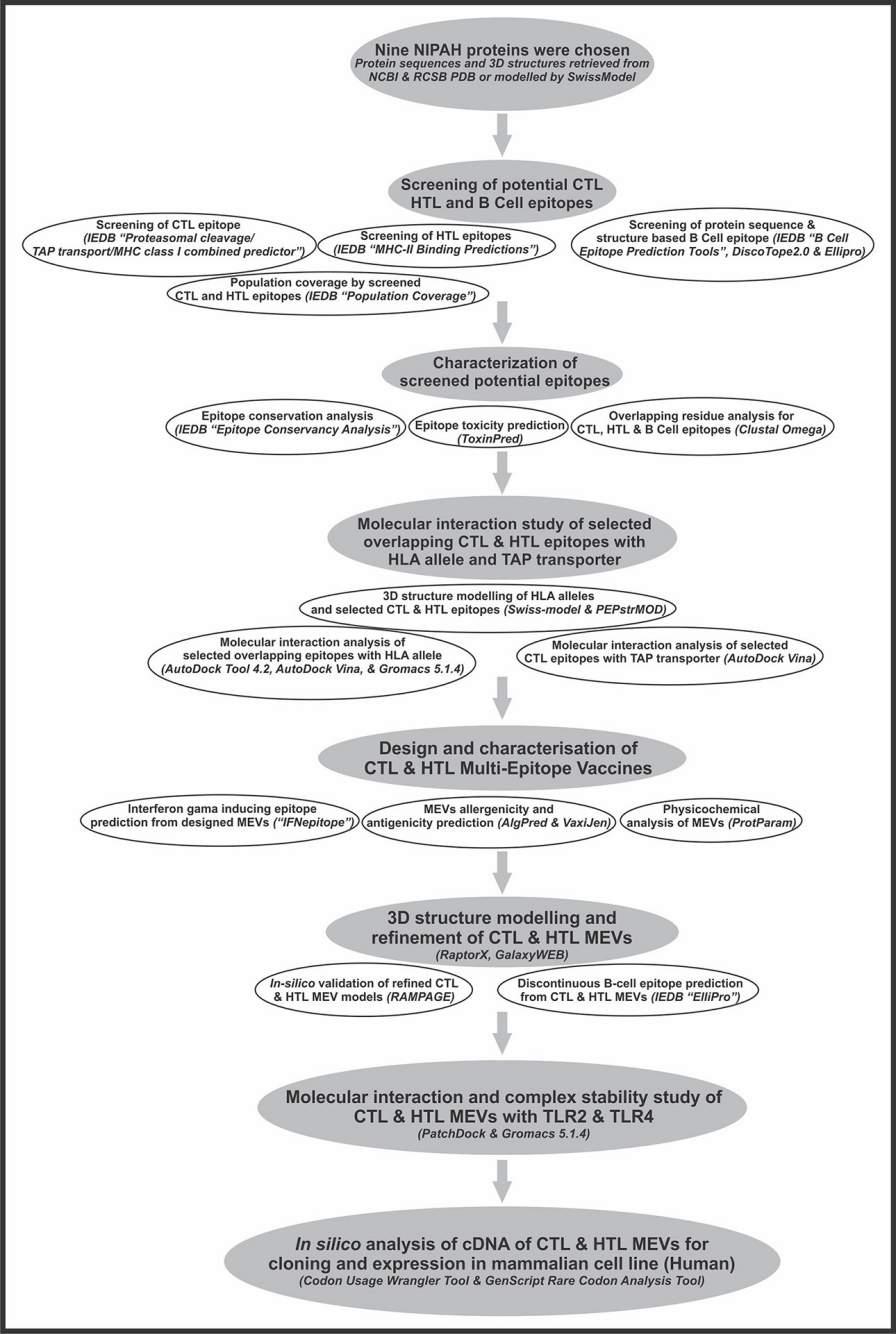
Workflow chart.

In the present study, we have designed two Multi-Epitope Vaccines (MEVs) composed of thoroughly screened most potential Cytotoxic T lymphocyte (CTL) epitopes and Helper T lymphocyte (HTL) epitopes derived from the nine NiV proteins (glycoprotein (gi-253559848); C protein (gi-1859635642); fusion protein (gi-13559813); matrix protein (gi-13559811); nucleoprotein (gi-1679387250); phosphoprotein (gi-1802790259); V protein (gi-1802790260); RNA polymerase (gi-15487370); W protein (gi-374256971). CTL and HTL epitopes would be the most potential vaccine candidates since they are responsible for cell-mediated immune response by their presentation on the surface of antigen presenting cells (APCs) by their respective Class I and II human leukocyte antigen (HLA) allele binders. Both the CTL and HTL epitopes chosen for MEV design also carry overlapping regions of linear B cell epitopes. Moreover, both the MEVs also carry potential discontinuous B cell epitopes as well as IFN-γ inducing epitopes in their tertiary structure model. Hence, both the designed MEVs carry potential to elicit cell-mediated as well as humoral immune response. Furthermore, both the MEVs were designed with the vaccine adjuvant human Beta-Defensin 2 and human Beta-Defensin 3 at their N and C termini to enhance the immune response [45,46]. Beta-defensins have considerable immunological adjuvant activity in general and, in particular the Beta-defensins 2 & 3 have been shown in previous studies to generate potent humoral immune responses when fused with B-cell lymphoma epitopes in mouse model [47–50]. Since, the pro-inflammatory mediators enhance the expression of Beta-defensins 2 & 3 in airway epithelial cells we chose Beta-defensins in our study. The shortlisted epitopes and adjuvants Beta-defensins 2 & 3 were linked together by short peptide linkers EAAAK and GGGGS to design MEVs against NiV [51–54]. The selected CTL and HTL epitopes were validated for their stable molecular interactions with their respective HLA alleles binders; for CTL epitopes, their molecular interaction with human Transporter associated with antigen processing (TAP) was also analyzed [55,56]. This analysis validated the CTL epitopes that get transported through the TAP cavity for their presentation on cell surface or not. The human TAP selectively transports cytosolic peptides into the lumen of the endoplasmic reticulum in an ATP-dependent manner [57]. Tertiary structure models of both the MEVs were generated, refined, and further docked with the human Toll-Like Receptor 3 (TLR3), which is an essential immuno-receptor in this pathway [58,59]. The nuclear localization of Nipah virus W protein inhibits the signaling pathway of TLR3 upon pathogenesis to suppress the TLR3 induced activation of the IFN response to eventually prevent relay of the warning signals to uninfected cells. TLR3 is preferentially expressed by human astrocytes of the central nervous system (CNS) upon infection. The NiV infection involves invasion of the neurons of CNS and hence causes infection in CNS. These studies indicate the importance of the TLR3 responses in immune response and hence TLR3 has been chosen to be studied for its stable binding with the designed Multi-Epitope Vaccines [60–63]. The complexes of CTL and HTL MEVs formed with the human TLR3 were further analyzed for their stable molecular interaction by a molecular dynamics simulation study. The cDNA of the designed MEVs were generated and analyzed for their high expression tendency in the mammalian host cell line (Human). Overall, from the present *in-silico* study, we may put forward the design of two MEVs, which qualify all the significant criterions for being a potential vaccine candidate against NiV infections. The corresponding workflow is shown in Fig 1.

### NiV proteins selected for potential epitope screening

In the present study, nine NiV proteins were used for epitope screening. They include C protein, Fusion glycoprotein (F), Glycoprotein (G), Matrix proteins (M), Nucleocapsid protein, Phosphoprotein, Polymerase, V protein and the W protein. The full-length protein sequences of NiV proteins were retrieved from the NCBI database (National Center for Biotechnology Information (https://www.ncbi.nlm.nih.gov/protein). A total of 161 protein sequences available at NCBI, belonging to different strains and origins of NiV, were retrieved. For structural based epitope screenings available tertiary structures of NiV proteins were retrieved from Protein Data Bank (PDB) (http://www.rcsb.org/pdb/home/home.do). For the NiV proteins with no structure available, homology models were generated by Swiss-model, (http://swissmodel.expasy.org/) [64].

### Screening of potential epitopes

#### T cell epitope prediction

*Screening of Cytotoxic T lymphocyte (CTL) Epitope*. The screening of Cytotoxic T lymphocyte epitopes was performed by the IEDB (Immune Epitope Database) tool “Proteasomal cleavage/TAP transport/MHC class I combined predictor” (http://tools.iedb.org/processing/) [65–67]. Proteasome cleavage score depends on the total amount of cleavage site in the protein. TAP score estimates an effective log -(IC50) values (half maximal inhibitory concentration (IC50)) for binding to TAP of a peptide or its N-terminal prolonged precursors. The MHC binding prediction score is -log(IC50) values for binding to MHC of a peptide [65–68]. The IC(50) (nM) value for each epitope and MHC allele binding pairs were also obtained by this IEDB tool. Immunogenicity of all the screened CTL epitopes was also obtained by using “MHC I Immunogenicity” tool of IEDB (http://tools.iedb.org/immunogenicity/) with all the parameters set to default [68].

*Screening of Helper T lymphocyte (HTL) Epitopes*. To identify the Helper T lymphocyte epitopes from NiV proteins, the IEDB tool “MHC-II Binding Predictions” (http://tools.iedb.org/mhcii/) was used. Peptides with IC50 values <50 nM are considered high affinity, <500 nM intermediate affinity and <5000 nM low affinity [69–72]. The tool generates percentile rank for each potential peptide.

*Population Coverage by T-cell epitopes*. The “Population Coverage” tool of IEDB (http://tools.iedb.org/population/) was used to elucidate the world human population coverage by the shortlisted 33 CTL and 38 HTL epitopes derived from nine NiV proteins [73].

#### B cell epitope prediction

*Sequence-based B Cell epitope prediction*. Protein sequence-based six different methods were utilized to screen linear B cell epitopes from nine different NiV proteins. These methods are available at “B Cell Epitope Prediction Tools” of IEDB server (http://tools.iedb.org/bcell/). The tool utilizes the propensity scale method as well as the physiochemical properties of the given antigenic sequence to screen potential epitopes using “Bepipred Linear Epitope Prediction”, “Chou & Fasman Beta-Turn Prediction”, “Emini Surface Accessibility Prediction”, “Karplus & Schulz Flexibility Prediction”, “Kolaskar & Tongaonkar Antigenicity” and “Parker Hydrophilicity Prediction” methods, available as sub-tools [74–79].

*Structure-based B cell epitope prediction*. The Ellipro (ElliPro: Antibody Epitope Prediction tool; http://tools.iedb.org/ellipro/) and the DiscoTope2.0 (DiscoTope: Structure-based Antibody Prediction tool; http://tools.iedb.org/discotope/) methods available at IEDB, were used to screen the linear and the discontinuous B cell epitopes [80,81]. The ElliPro predicts the epitope on the basis of location of residue in the protein’s 3D structure. Predicted epitope is given a score which is defined as averaged PI (Protrusion Index) value over epitope residues. The residues lying outside of an ellipsoid covering 90% of the inner core protein residues score highest Protrusion Index (PI) of 0.9 which indicate largely surfaced residues and so potential target as an epitope. Further the Discotope 2.0 method is based on the “contact number” of the residue’s Cα carbon atom as well as on the propensity of a given residue to be a part of an epitope. The residue “contact number” is the number of Cα atoms in the antigen within a distance of 10 Å of the particular residue’s Cα atom. A low contact number would indicate the residue being close to the surface or in protruding regions of the antigen’s structures and hence a potential target as an epitope.

### Characterization of potential epitopes

#### Epitope conservation analysis

The shortlisted CTL, HTL and B cell epitopes screened from nine NiV proteins were analyzed for the conservancy of their amino acid sequence by “Epitope Conservancy Analysis” tool (http://tools.iedb.org/conservancy/) of IEDB, against the respective source protein sequences of NiV proteins retrieved from the NCBI protein database [82].

#### Epitope toxicity prediction

The tool ToxinPred (http://crdd.osdd.net/raghava/toxinpred/multi_submit.php) was used to analyze the toxicity of shortlisted CTL, HTL and B cell epitopes. The tool allows to identifying highly toxic or non-toxic short peptides. The toxicity check analysis was done by the “SVM (Swiss-Prot) based” (support vector machine) method. utilizing dataset of 1805 sequences as positive, 3593 negative sequences from Swissprot as well as an alternative dataset comprises the same 1805 positive sequences and 12541 negative sequences from TrEMBLE [83].

#### Overlapping residue analysis

The overlapping residue analysis for the shortlisted CTL, HTL and the B cell linear epitopes was performed by the Multiple Sequence Alignment (MSA) analysis by Clustal Omega tool (https://www.ebi.ac.uk/Tools/msa/clustalo/) of EBI (European Bioinformatics Institute) [84]. The Clustal Omega multiple sequence alignment tool virtually aligns any number of protein sequences and delivers an accurate alignment. The overlapping epitopes were shortlisted for further in silico validation.

#### Tertiary structure modeling of epitopes and HLA allele binders

The Swiss-model was used for homology modelling of the HLA class I and II allele binders of shortlisted epitopes [64]. The amino acid sequence of the HLA allele binders were retrieved from Immuno Polymorphism Database (IPD-IMGT/HLA) (https://www.ebi.ac.uk/ipd/imgt/hla/allele.html). Templates for homology modelling were chosen on the basis of highest amino acid sequence similarity. All the HLA allele models were further validated by their QMEAN value. The QMEAN value gives a composite quality estimate involving both global as well as local analysis of the model [85]. Generated models having acceptable QMEAN value (cutoff -4.0) were chosen for further studies.

The “Natural Peptides Module for Beginners” module of PEPstrMOD (http://osddlinux.osdd.net/raghava/pepstrmod/nat_ss.php) was utilized to generate tertiary structures for the selected CTL and HTL epitopes [86]. The time window for simulation was set to 100 picoseconds (ps) in a vacuum environment.

#### Molecular interaction analysis of epitopes with HLA alleles

The AutoDock 4.2 (ADT) and the AutoDock Vina were used for *in-silico* molecular docking study of the selected CTL and HTL epitopes with their respective HLA class I and II allele binders [87,88]. The generated docked complexes were studied for their stable nature by molecular dynamics simulation. MD simulation was performed by the Gromacs 5.1.4 using the Optimized Potential for Liquid Simulations—all-atom force field (OPLS-AA) [89,90].

#### Molecular interaction analysis of epitopes with TAP transporter

Molecular interaction study of the shortlisted CTL epitopes with the TAP transporter cavity was performed by molecular docking using AutoDock Vina [87,88]. As structural model the cryo-EM structure of TAP transporter (PDB ID: 5u1d) after removing the antigen from TAP cavity of the original structure [56] was used for epitope-TAP interaction study. TAP transporter plays an important role in the presentation of CTL epitopes. Following the immuno-proteasomal processing of xenobiotic proteins, the fragmented peptides get transported to the endoplasmic reticulum (ER) through the TAP transporter [91].

### Characterization of multi-epitope vaccines

#### Interferon gamma inducing epitope prediction

From the designed amino acid sequence of both the MEVs potential interferon gamma (IFN-γ) epitopes were screened via the “IFNepitope” server (http://crdd.osdd.net/raghava/ifnepitope/scan.php) using the “Motif and SVM hybrid”, (MERCI: Motif-EmeRging and with Classes-Identification, and SVM: support vector machine) method [92,93].

#### MEVs allergenicity and antigenicity prediction

The designed MEVs were further analyzed for allergenicity and antigenicity prediction by utilizing the AlgPred (http://crdd.osdd.net/raghava/algpred/submission.html) and the Vaxijen (http://www.ddg-pharmfac.net/vaxijen/VaxiJen/VaxiJen.html) tools respectively [94,95].

#### Physicochemical property analysis of designed MEVs

The ProtParam (https://web.expasy.org/protparam/) tool was utilized to analyze the physiochemical properties of the designed CTL and HTL MEVs [96]. The ProtParam analysis performs an empirical investigation for the given query amino acid sequence.

#### Tertiary structure modelling and refinement of MEVs

The tertiary structure of the designed CTL and HTL MEVs were calculated by homology modelling utilizing the RaptorX structure prediction tool (http://raptorx.uchicago.edu/StructurePrediction/predict/) [97–100].

The refinement of both the generated MEV models was performed by ModRefiner (https://zhanglab.ccmb.med.umich.edu/ModRefiner/) and GalaxyRefine tool (http://galaxy.seoklab.org/cgi-bin/submit.cgi?type=REFINE) [101,102]. Modrefiner improves the structural accuracy of the protein model and provide significant improvement in the physical quality of the structures [101]. The GalaxyRefine tool generates reliable core structures of protein by using an optimization-based refinement method [100,103].

*Validation of CTL and HTL MEVs refined models*. Both the refined CTL and HTL MEV 3D models were further validated by RAMPAGE analysis tool (http://mordred.bioc.cam.ac.uk/~rapper/rampage.php) [104,105]. The generated Ramachandran plots for the MEV models show the sterically allowed and disallowed residues along with their dihedral psi (ψ) and phi (φ) angles.

*Discontinuous B-cell epitope prediction from MEVs*. Both the generated tertiary models of designed CTL and HTL MEVs were subjected to discontinuous B cell epitopes prediction. The structure-based discontinuous B cell epitopes were screened from both the MEV models by utilizing the ElliPro method as described earlier [80].

### Molecular interaction analysis of MEVs with TLR3

Molecular interaction analysis of both the designed MEVs with TLR3, was performed by molecular docking and molecular dynamics simulation. Molecular docking was performed by PatchDock server (http://bioinfo3d.cs.tau.ac.il/PatchDock/) [106–108]. PatchDock utilizes algorithm for unbound (real life) docking of molecules for protein-protein. The algorithm carries out the rigid docking, with the surface variability/flexibility implicitly addressed through liberal intermolecular penetration. The algorithm focuses on the (i) initial molecular surface fitting on localized, curvature based surface patches (ii) use of Geometric Hashing and Pose Clustering for initial transformation detection (iii) computation of shape complementarity utilizing the Distance Transform (iv) efficient steric clash detection and geometric fit scoring based on a multi-resolution shape representation and (v) utilization of biological information by focusing on hot spot rich surface patches [106–108]. For molecular docking, the 3D structure of the human TLR3 ectodomain (ECD) was retrieved from the PDB databank (PDB ID: 2A0Z). The Residue interactions across interface of the CTL and HTL MEVs in complex with TLR3(ECD) were analyzed by PDBsum [109]. The Protein-protein interface (Prot-Prot) application of PDBsum was utilized to generate the residue to residue interaction of MEVs and TLR3(ECD). Further, the molecular dynamics simulation study of the MEVs-TLR3 complexes was performed by YASARA tool (Yet Another Scientific Artificial Reality Application) [110]. The MD simulations studies were carried out in an explicit water environment in a dodecahedron simulation box at a stabilized temperature of 298K, the pressure of 1atm and pH 7.4, with periodic cell boundary condition. The solvated systems were neutralized with counter ions (NaCl) (concentration 0.9 M). The AMBER14 force field was used on the systems during MD simulation [111]. The Long-range electrostatic energy and forces were calculated using particle-mesh-based Ewald method [112]. The solvated structures were energy minimized by the steepest descent method at a temperature of 298K and a stable pressure of 1 atm. Further, the complexes were equilibrated for period of 1 ns. After equilibration, a production MD simulation was run for 100 ns at a stable temperature and pressure and time frames were saved at every 100 ps, for each MD simulations. The RMSD and RMSF values for Cα, Backbone and all the atoms of all the CTL and HTL MPVs in complex with TLR3 were analyzed for each MD simulation study.

### *In-silico* analysis of MEVs for cloning and expression

Complementary DNA of both the MEVs, codon optimized for expression in mammalian cells were generated by Java Codon Adaptation Tool (http://www.jcat.de/). The generated cDNA was further analyzed with the GenScript Rare Codon Analysis Tool (https://www.genscript.com/tools/rare-codon-analysis) which calculates the GC content, Codon Adaptation Index (CAI) and the Tandem rare codon frequency for a given cDNA [113–115].

## Result and discussion

### Screening of potential epitopes

#### Screening of T cell and B cell epitopes

The Cytotoxic T lymphocyte (CTL) epitopes were screened and shortlisted according to their highest “Total Score”, low IC(50) (nM) value for epitope-HLA class I allele complexes, and epitopes with the larger number of the HLA class I allele binders. The “Total Score” is a combined score of the proteasome, MHC, TAP (N-terminal interaction), and processing analysis scores. It is calculated by using the combination of six different methods viz. Consensus, NN-align, SMM-align, Combinatorial library, Sturniolo and NetMHCIIpan [65–68]. Overall an increasing “Total Score” indicates a higher propensity of the epitope to get processed thru HLA allele, TAP cavity and proteasome and presented as a potential epitope candidate. Further, the epitopes having high, intermediate, and least affinity of binding to their HLA allele binders have IC50 values < 50 nM, < 500 nM and < 5000 nM respectively, indicating epitope-HLA allele affinity of binding. The immunogenicity of the shortlisted CTL epitopes was also determined; the higher immunogenicity score indicates increasing immunogenic potential of the given epitope (S1 and S2 Tables in S1 File). The immunogenicity of a given peptide-MHC (pMHC) complex is determined by the physiochemical properties of constituting 1^st^, 2^nd^, and C-terminus amino acids of the given screened epitope. Further, in total 33 CD8+ T cell epitopes were finally chosen scoring high for the above mentioned criterion. From this list 10 CTL epitopes (Fusion Protein: FALSNGVLF; Glycoprotein: TVYHCSAVY; Nucleocapsid: YPALALNEF; Phosphoprotein: VSDAKMLSY; Polymerase: YPECNNILF, FPVMGNRIY, AEFFSFFRTF, IPFLFLSAY, ETDDYNGIY, SQNLLVTSY) show a match with previous studies [35–38], indicating consensus of epitope screening by different approaches and methods (S1 Table in S1 File). The 33 shortlisted CD8+ T cell epitopes identified here for the MEV design were neither published before nor used for vaccine design. In contrast to previous studies focusing mostly on four protein targets we have identified the epitopes from nine different NiV proteins, thus targeting entire proteome of the virus. The MEVs reported here have a strong potential for developing an efficient vaccine candidate.

The screening of helper T lymphocyte (HTL) epitopes from nine different proteins of NiV was performed on the basis of “Percentile rank”. The percentile rank is generated by the combination of three different methods viz. combinatorial library, SMM_align and Sturniolo and by comparing against other random five million 15-mer peptides of SWISSPROT database. The rank from the consensus of all three methods was generated by the median percentile rank of the three methods. A smaller value of percentile rank indicates higher affinity of the peptide with its respective HLA allele binders. In total 38 epitopes out from initial screening were chosen on the basis that they had highest percentile rank and can bind to largest number of different HLA class II allele (S3 and S4 Tables in S1 File). The identified 38 HTL epitopes have not been reported before and used for first time to design MEVs. They are also derived from all the nine proteins of NiV and hence provide greater potential to the Multi-Epitope vaccine candidate.

**Fig 2:**
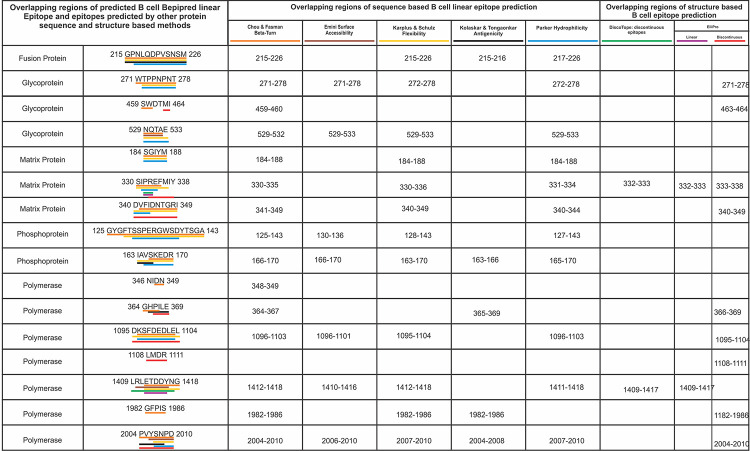
Overlapping regions amongst the linear B cell epitopes predicted by the BepiPred method and other seven different B cell epitopes prediction methods. B cell epitopes predicted by the BepiPred method and other different protein sequence based (Chou.., Emini.., Karplus.., Kolaskar.., and Parker..) and protein structure based (DiscoTope and ElliPro) prediction methods were found to have significant consensus. Consensus overlapping regions of BepiPred epitopes are underlined by the different color, corresponding to respective prediction method.

We screened 116 B Cell linear epitope from nine different NiV proteins with epitope length of at least four amino acids utilizing Bepipred Linear Epitope Prediction tool. The screening is based on several different parameters such as hydrophilicity, flexibility, accessibility, turns, exposed surface, polarity and antigenic propensity of the peptides which is correlated with their location in the protein. This also allows the search for continuous epitopes prediction from protein sequence. The prediction is based on the propensity scales for each of the 20 natural amino acids. When computing the score for a given residue I, the amino acids in an interval of the chosen length, centered around residue i, are considered. B cell epitopes predicted by another five different screening tools as mentioned in methods section, based on different physiochemical properties were found to have significant consensus with the epitope amino acid sequences predicted by Bepipred Linear Epitope Prediction. Further, 16 out of the 116 epitopes were shortlisted having a length of 4 to 19 amino acids (S5 Table in S1 File, Fig 2). One of these 16 B cell epitopes (Matrix Protein: SIPREFMIY) matches with a previous study [39], indicating epitope screening consensus using different approaches (S5 Table in S1 File). Further, the structure-based discontinuous and linear epitopes were also screened. To screen structure base B cell epitopes the ElliPro and Discotope [80,81] methods were used. For both the methods the prediction models or available structures in PDB database for nine NiV proteins were used (S6 Table in S1 File).

**Fig 3:**
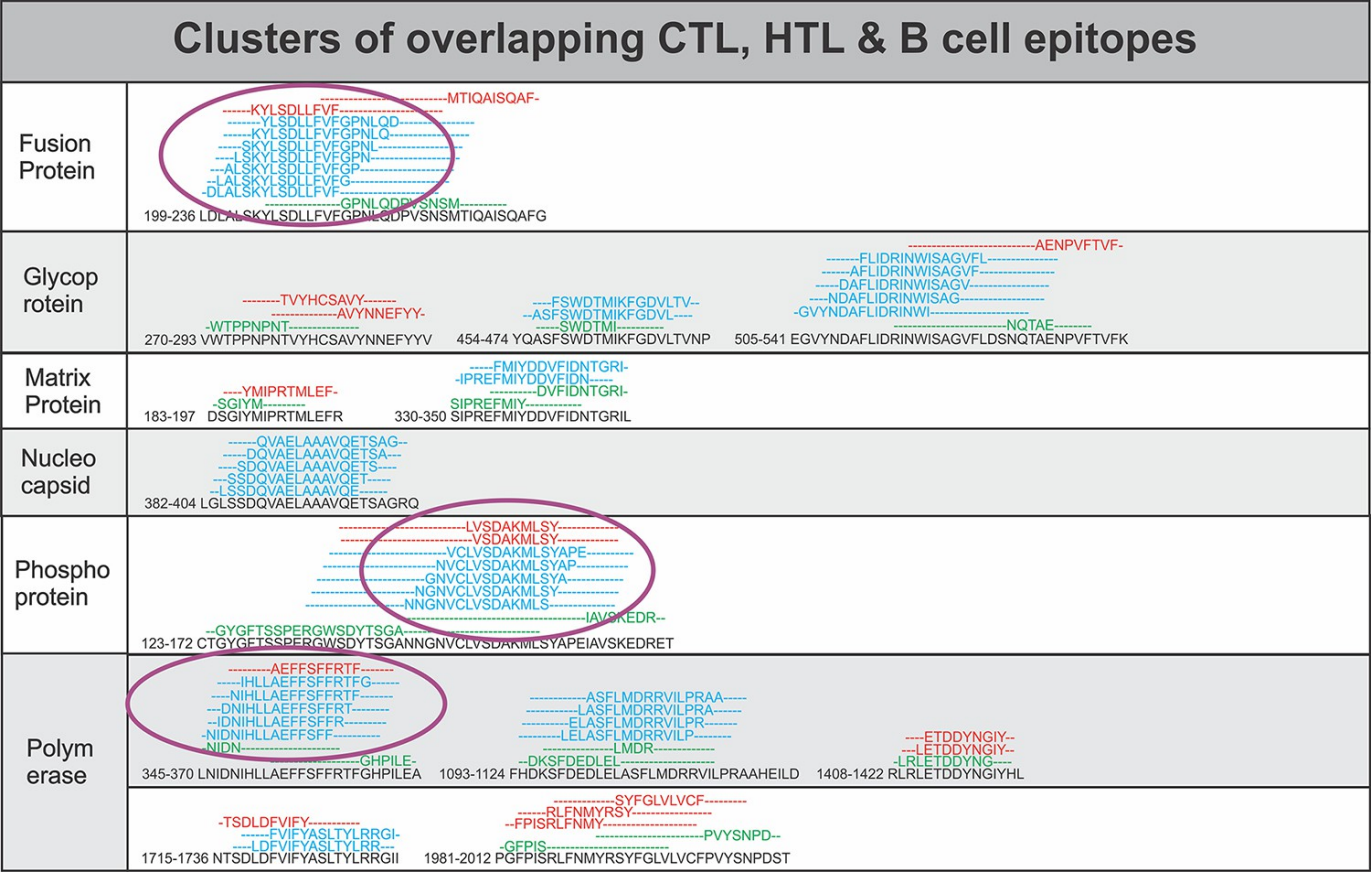
Overlapping CTL, HTL and B cell epitopes. Multiple sequence alignment performed by Clustal Omega at EBI to identify the consensus overlapping regions of CTL (red), HTL (blue) and B cell epitopes (green) amongst shortlisted epitopes. Epitopes with overlapping region amongst all the three types of epitopes (CTL, HTL and B Cell epitopes), epitopes with full sequence overlap and epitopes with the highest number of HLA allele binders were selected for further studies (encircled).

These screened T cell epitopes have shown a significant consensus of overlapping amino acid sequence with the predicted B cell linear epitopes (S5 Table in S1 File, Fig 2). These results indicate that the shortlisted B Cell Epitopes have a high potential to elicit a strong immune response in humans. Further analysis of the amino acid sequence overlap amongst the shortlisted CTL, HTL and B cell epitopes from nine different NiV proteins was also performed. Our analysis showed that several epitopes of CTL, HTL and B cell have overlapping amino acid sequence. Besides a few shorter identical sequences the majority of the CTL, HTL and B cell epitopes have several amino acids overlap as shown in Fig 3. This analysis indicates that most of the CTL, HTL and B cell epitopes arise from the common region of the protein. The overlap amongst epitopes also indicates that the methods used to screen CTL, HTL and B Cell epitopes have a consensus in their results, further this raises the propensity of the epitopes to be high in potential to cause immune response. Those CTL, HTL and B Cell epitopes that showed overlap with each other were chosen for detailed study (Fig 3, S1 and S3 Tables in S1 File). Taken together, we have found that our approach resulted in overlapping epitopes for NiV that might be useful to design novel MEVs against NiV.

### Population coverage by CTL and HTL epitopes

Since, the MHC types are expressed at dramatically different frequencies in different ethnicities, the population coverage by the shortlisted epitopes was tested, in particular involving countries from South Asia, East Asia, Northeast Asia and the Middle East. The differentially distributed HLA alleles provide an opportunity to analyze the population coverage by the epitopes candidates targeting or having affinity to bind to different HLA alleles. A given epitope will elicit a response only in individuals that express an HLA allele molecule, which is capable of binding to that particular epitope. This denominated HLA restriction of T cell responses and the MHC polymorphism provide basis for population coverage study. Hence, a vaccine with optimized population coverage could be of greater relevance in fighting NiV caused disease. From this study, we concluded that the combined clinical administration of multiple-epitopes involving both the CTL and the HTL epitopes would have a cumulative percent of world (S7 Table in S1 File) population coverage as high as 97.88%.

### Characterization and structural based validation of epitopes

#### Conservancy, toxicity and interaction with HLA & TAP molecules

The amino acid sequence conservation analysis of the shortlisted 33 CTL, 38 HTL and 16 B cell epitopes show that the amino acid sequence conservancy of CTL, HTL and B cell epitopes amongst all retrieved NiV protein sequences is mostly 100%, as shown in S1, S3 and S5 Tables in S1 File. The epitope conservancy is the percentage of protein sequences that contain that particular epitope. The analysis was done against full length protein sequences retrieved from the NCBI protein database, all the available NiV full length proteins sequences were retrieved for conservancy analysis.

Next, we analyzed the predicted toxicity of all the shortlisted CTL, HTL and B Cell epitopes. The ToxinPred study indicated the non-toxic nature of all the shortlisted epitopes as summarized in S1, S3 and S5 Tabels in S1 File. The toxicity is determined by the physiochemical properties and Swiss-Prot based SVM (support vector machine) score of the epitopes comparative to the known epitope dataset. The dataset of known 1805 positive (toxic) sequences, 3593 negative (non-toxic) sequences from Swissprot as well as an additional dataset comprising 1805 positive sequences and 12541 negative sequences from TrEMBLE were utilized.

**Fig 4:**
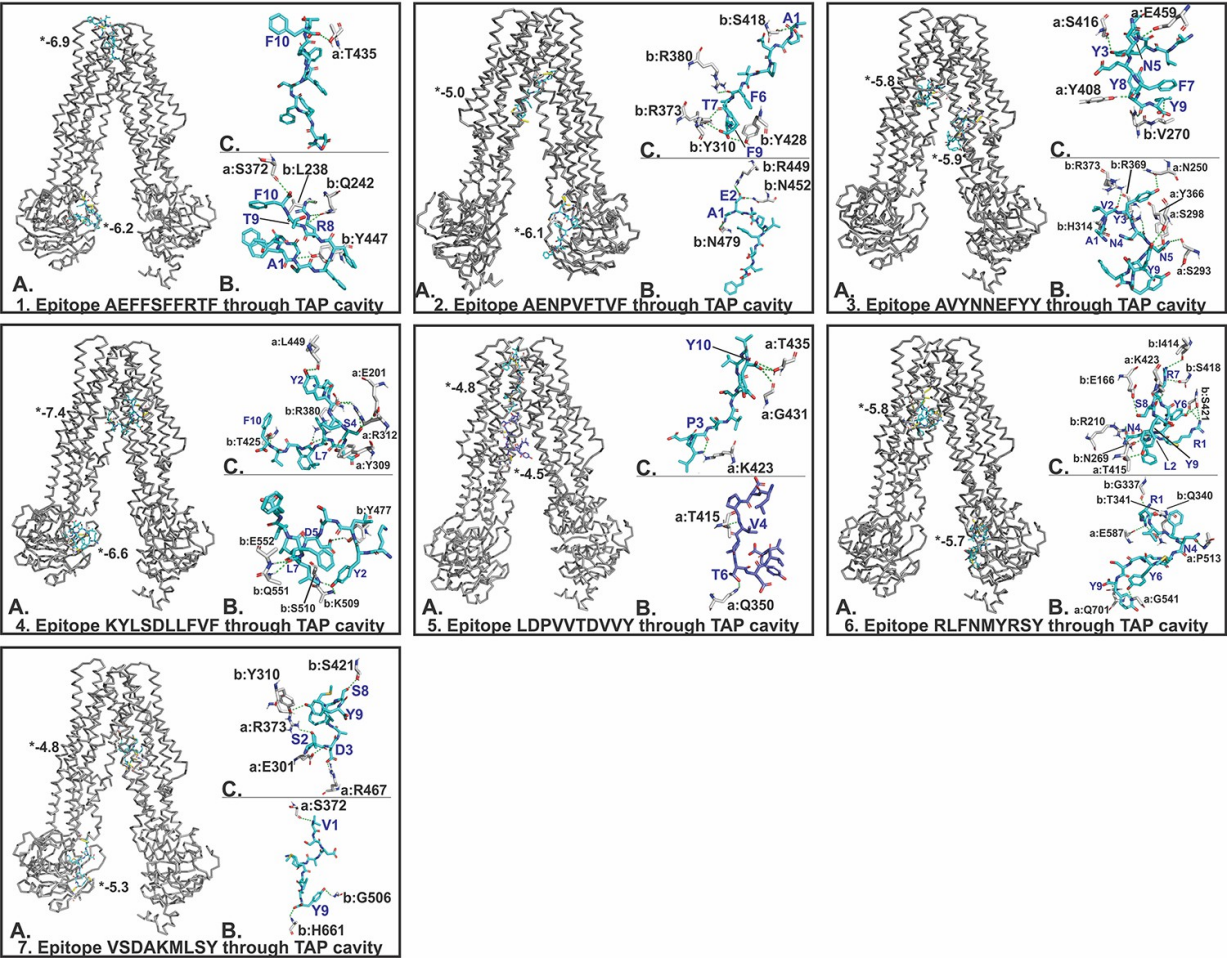
Molecular docking analysis of CTL epitopes within the TAP transporter cavity. Molecular interaction of the seven selected CTL epitopes (cyan sticks) with the TAP cavity (gray ribbon/sticks) is shown in detail. Panel **(A)** shows the binding of epitope at two different sites within TAP cavity, panel **(B)** and **(C)** show detailed molecular interaction between epitopes and TAP cavity; (a, b) show chain A and B of TAP transporter. H-bonds are highlighted as yellow dots. (*) Indicates binding energy, shown in kcal/mol.

The molecular docking study of the shortlisted CTL and HTL epitopes with their respective HLA class I and II allele binders was performed. The tertiary structural model of binding HLA alleles were generated by homology modeling using SwissModel. The best model with acceptable QMEAN values (cutoff -4.0), were chosen for docking studies with epitopes (S8 Table in S1 File). The QMEAN values indicating accuracy of models as a composite quality estimate involving both global as well as local analysis of the model. This analysis was done with HLA allele as rigid and epitopes as flexible interaction molecule having rotatable bonds. The molecular docking study revealed for all epitopes significant molecular interactions with their HLA allele binders having low binding energies and multiple hydrogen bonds formed (S1 Fig in S1 File). The B-factor analysis of all the epitope-HLA allele complexes indicated most of the complex regions to be stable (blue) with a very small region being acceptably fluctuating (yellow and orange) (VIBGYOR color presentation) (S2 Fig in S1 File). We also performed the analysis of the prevalence of amino acids of epitopes binding to the HLA allele molecules. It showed that the amino acid Phenylalanine (F), Tyrosine (Y), Serine (S) and Asparagine (N) of the epitopes were the most prevalent ones to form hydrogen bonds with the HLA class I and I alleles molecules (S9 Table in S1 File). Amongst those prevalent amino acids the residues Tyrosine, Serine and Asparagine are polar in nature and are frequently found on the surface of the antigenic proteins, indicating that they are directly interacting with HLA molecules. Phenylalanine with its more a hydrophobic side chain is often found in intermolecular interaction between HLA molecule and epitopes. Further, the stability of the obtained docking complexes was tested by molecular dynamics (MD) simulation studies. MD simulations were performed over a time interval of 20 ns at the invariable temperature of ~ 300 K and at invariable pressure of ~ 1 bar. All the complexes showed reasonably invariant effective root mean square deviation (RMSD) value ~ 1 Å indicating the stable nature of the tested epitope-HLA allele complexes (S3 Fig in S1 File). Moreover, the reasonably invariant Rg (radius of gyration) of the complexes, throughout the MD simulation (S4 Fig in S1 File), and the root mean square fluctuation (RMSF) for all the atoms of the complexes (S5 Fig in S1 File) again indicate the stable nature of the epitopes and HLA allele complexes. Hereafter the chosen CTL epitopes were analyzed for their molecular interaction with the TAP transporter cavity. The molecular docking interaction analysis showed a strong molecular interaction with low binding energy and several hydrogen bonds formed at different sites of the TAP transporter cavity. Two sites of interaction were of particular interest, one located near the cytoplasmic end and the other in the vicinity of the ER lumen (Fig 4). Our study confirms the transportation feasibility of the chosen CTL epitopes from the cytoplasm into the ER lumen which is essential for the representation of peptides by the HLA allele molecules on the surface of antigen presenting cells.

### Design and characterization of multi-epitope vaccines

#### Allergenicity, antigenicity, physicochemical properties, MEV tertiary structure, IFN-γ inducing and discontinuous B-cell epitopes and cDNA analysis

**Fig 5:**
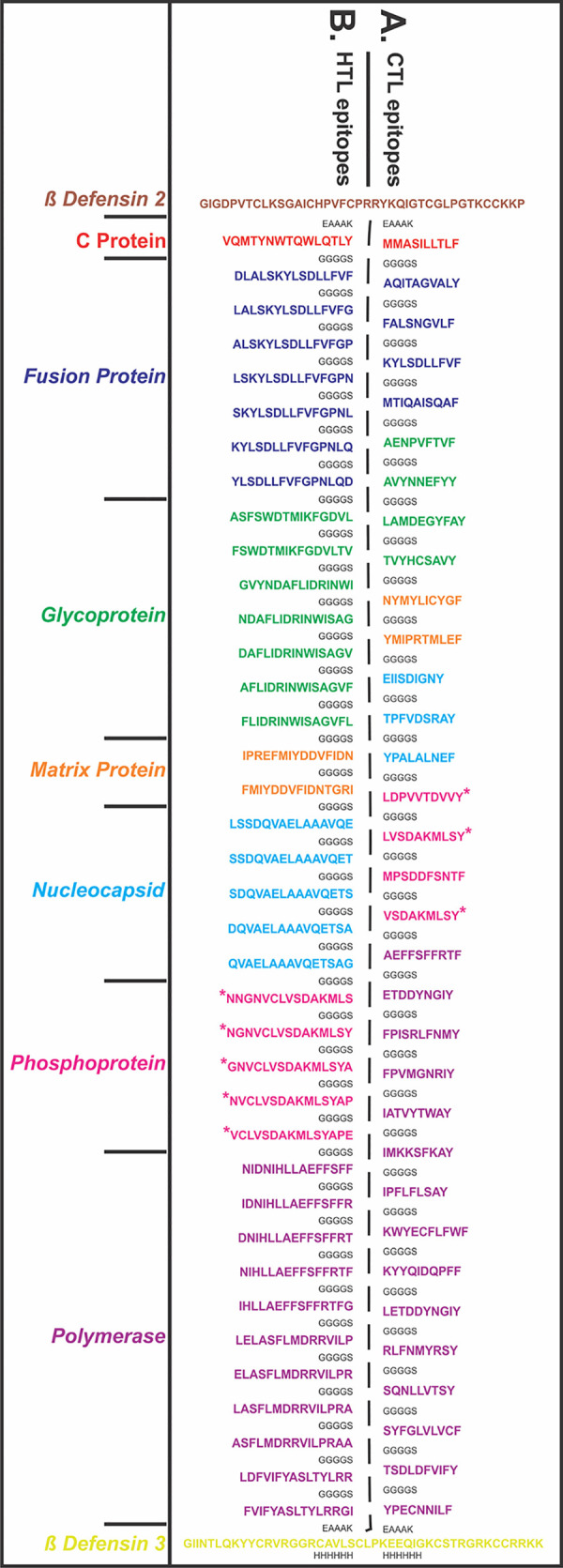
Design of Multi-Epitope Vaccine (MEVs). **(A)** CTL and **(B)** HTL epitopes were linked by the short peptide linker ‘GGGGS’. Human Beta-Defensin 2 and Beta-Defensin 3 were used as an adjuvant at the N and C terminals respectively. The short peptide EAAAK was used to link the Beta-Defensin 2 and Beta-Defensin 3. Epitopes from different proteins were highlighted with different colors and the C terminal 6xHis is designated as His tag. *Indicates the epitopes common to Phosphoprotein, V Protein and W protein, dues to protein sequence similarity.

The chosen 33 CTL and 38 HTL epitopes were utilized to design CTL and HTL Multi-Epitope Vaccines. Short peptides EAAAK and GGGGS were used as rigid and flexible linkers respectively (Fig 5). The GGGGS linker provides proper conformational flexibility to the vaccine tertiary structure and hence facilitates stable conformation to the vaccine. The EAAAK linker facilitates in domain formation and hence facilitates the vaccine to obtain its final stable structure [45–54]. The human Beta-defensin 2 (hBD-2) (PDB ID: 1FD3, Sequence: GIGDPVTCLKSGAICHPVFCPRRYKQIGTCGLPGTKCCKKP) and the human Beta-Defensin 3 (hBD-3) (PDB ID: 1KJ6, Sequence: GIINTLQKYYCRVRGGRCAVLSCLPKEEQIGKCSTRGRKCCRRKK) were used as adjuvants in the design of both the MEVs at N and C terminals respectively [45–54]. human Beta-defensins have an important role in the chemotactic activity memory T cells, immature dendritic cells and monocytes and they are involved in the degranulation of mast cells. Due to the important role of hBDs in immune response enhancement, hBDs have been chosen and utilized as adjuvants for the MEV designs.

Allergenicity and antigenicity of the designed MEVs was also analyzed by using the program AlgPred. Both the CTL and HTL MEVs were found to have no allergenic potential (NON-ALLERGEN) with test scores of -0.612 and -0.935 respectively while default threshold value being -0.4. The AlgPred prediction compares the sequence similarity between known epitopes with any region of the submitted protein. For the screening of allergenicity, the Swiss-prot dataset consisting of 101725 non-allergens and 323 allergens was utilized. The prediction indicated no allergy potential for humans of the designed MEVs. The CTL and HTL MEVs were also confirmed by VaxiJen as antigens with prediction scores of 0.4447 and 0.4836 respectively, while the default threshold value for viral proteins is 0.4. The VaxiJen performs an alignment-free approach, solely based on comparing the physicochemical properties of the query amino acid sequence with the Swiss-prot dataset. For prediction of antigenicity, the bacterial, viral and the tumor protein datasets is used to derive models for the prediction of whole protein antigenicity. Every dataset consists of 100 known antigens and 100 non-antigens. The prediction indicated that all the designed MEVs are antigenic for humans. Both MEVs were also predicted to be non-allergic but antigenic in nature for human and so have promising properties for vaccine development.

Physiochemical properties of both the MEVs were analyzed. Therefore, ProtParam calculations were performed for the designed MEVs. The CTL MEV is composed of 576 amino acids, has a molecular weight of 58.57 kDa and a theoretical pI of 8.19. The expected half-life of the CTL MEV in *E*.*coli*, yeast and mammalian reticulocytes were predicted with 10 h, 20 min, and 30 h respectively; the aliphatic index has a predicted value of 58.42, and the grand average of hydropathicity (GRAVY) of was found to be -0.010, both indicating the globular and hydrophilic nature of the CTL MEV. The instability index score of the CTL MEV was 48.03 indicating its stable folding under native conditions. Similarly, the ProtParam analysis of the HTL MEV calculated for the 857 amino acids, a molecular weight of 87.62 kDa and a theoretical pI of 5.99. The expected half-life was predicted to be 10 h, 20 min and 30 h in *E*.*coli*, yeast and mammalian reticulocytes, respectively. The aliphatic index was calculated as 82.99, and the grand average of hydropathicity (GRAVY) was found to be 0.188, indicating that HTL MEV has a globular and hydrophilic nature. The instability index of the HTL MEV was 45.66 indicating its stable nature. Overall, the physiochemical properties of the proposed MEVs indicate that both genes might be expressed in their native forms adopting a stable fold essential for future protein purification using standard protocols.

**Fig 6:**
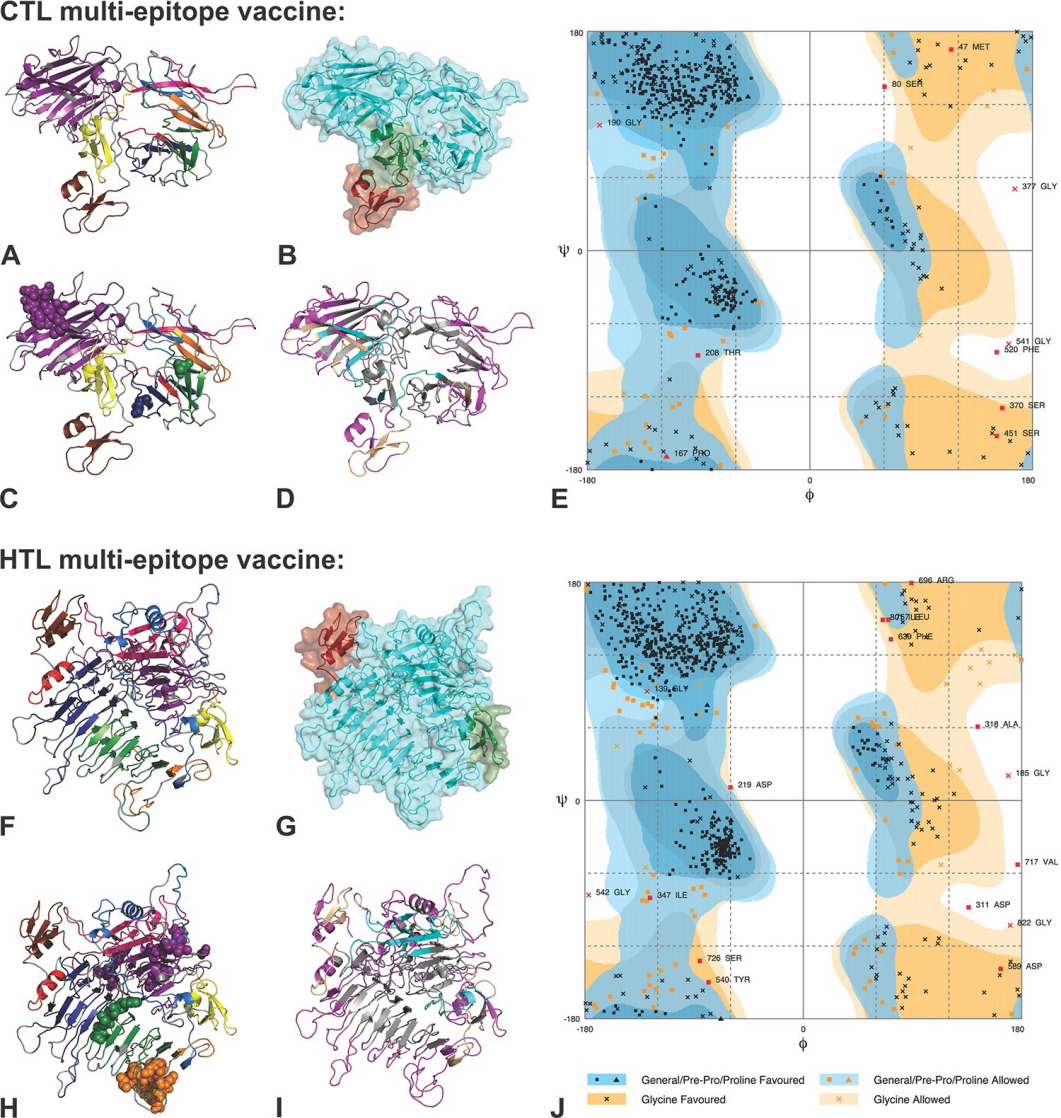
Tertiary structure modelling of CTL and HTL Multi-Epitope Vaccines. **(A) & (F)**: Tertiary structural models of CTL and HTL MEVs showing epitopes in different colors corresponding to the epitopes color as shown in Fig 5. **(B) & (G)**: Show the different domains of CTL and HTL MEVs. **(C) & (H)**: The overlapping linear B cell epitope region present in CTL and HTL MEVs, shown by spheres. **(D) & (I):** From the CTL and HTL MEVs, the INF-γ inducing epitopes are shown in cyan, discontinuous B Cell epitopes are shown in magenta and the region common amongst INF-γ and discontinuous B Cell epitopes are shown in wheat color. **(E) & (J)**: RAMPAGE analysis of the refined CTL and HTL MEV models.

Further, tertiary structural models were generated for both the CTL and HTL MEVs by utilizing the RaptorX modelling tool (Fig 6) predicting template-based secondary and tertiary structures of protein. The prediction also considers protein-protein contacts, solvent accessibility, presence of disordered regions, binding sites and similar homologs in the Protein Data Bank (PDB) for the given protein sequence.

The obtained CTL MEV model has 6% helix, 27% β-sheet, 66% coil content with 23% exposed, 39% medium and 36% buried structural elements. The structural model of the CTL MEV has three domains ranging from amino acid 1 to 46 (1^st^ domain, template-1fd3:A), 47 to 520 (2^nd^ domain, templates-1yrzA, 1y7bA, 5jozA, 3zxjA, 5z5dA) and 521 to 576 (3^rd^ domain, template-1kj6:A) (Fig 6B). Similarly, the RaptorX 3D model for the HTL MEV has 21% helix, 22% β-sheet, 56% coil content with 24% exposed, 39% medium and 36% buried amino acids. The HTL MEV has also three predicted domains ranging from amino acid 1 to 46 (1^st^ domain, template-1fd3:A), 47 to 801 (2^nd^ domain, templates-5m5zA, 3eqnA), 802 to 857 (3^rd^ domain, template-1kj6:A) (Fig 6G). The P-Value for the best template based CTL and HTL MEV homology models were 2.79e-04 and 5.99e-03 respectively. Good quality, mostly alpha proteins have a P-value of less than 10^−3^ and that of mostly beta proteins has a P-value of less than 10^−4^. The P-value (probability score) indicates a relative score of the generated strucutres in terms of modeling error, combining the global distance test (GDT) and the un-normalized global distance test (uGDT) indicating the error involved at each residue. The smaller the P-value indicates greater quality of a predicted model. Here the P-values for both MEVs indicate protein models with good quality. Since for the CTL and HTL MEV design, the CTL and HTL epitopes used also show overlapping common regions with the linear B cell epitopes (Fig 2), both the generated CTL and HTL MEV models also carry the overlapping regions of linear B Cell epitopes as shown in Figs 5H and 6C.

The generated CTL and HTL 3D models were further refined using ModRefiner to fix structural gaps followed by GalaxyRefine refinement. Modrefiner improves the physical realism and structural accuracy of the model using a two-step atomic-level energy minimization employing atomic-level, high-resolution protein tertiary structure refinement. Both the side-chain and the backbone atoms are kept flexible during the structure refinement simulations complying with a composite knowledge based force field. The further refinement was performed with the GalaxyRefine program optimizing the initial tertiary model by repeated structure perturbation as well as by utilizing the subsequent structural relaxation by the molecular dynamics simulation. The MolProbity score generated for a given refined model indicates the log-weighted combination of the clash score (the number of atomic clashes per 1000 atoms), the Ramachandran favored backbone torsion angles and the percentage of bad side-chain rotamers (the percentages of rotamer outliers). The ‘GDT-HA’ (Global Distance Test-High Accuracy) generated by the tool indicates the backbone structure accuracy; ‘RMSD’ (Root mean Square Deviation) indicates the overall structural deviation in refined model from the initial model and the ‘Rama favored’ indicates percentage of Ramachandran favored residues.

The first refinement with ModRefiner showed a TM-score of 0.9703 and 0.8934 for the CTL and HTL MEV models, respectively, indicating high similarity of the refined and the input model(identical start and refined model will give a TM-Score of one). Scores for the second CTL and HTL MEV model refinement calculated with Galaxy are given in S10 Table in S1 File. The different score indicated that after refinement, all the mentioned parameters were significantly improved in comparison to the initial CTL and HTL MEV models. Hence, the refined models of both the MEVs are acceptable for further experiments.

The obtained models were analyzed with the RAMPAGE analysis tool after refinement. The refined CTL MEV model has 91.5% residues in favored regions and the refined HTL MEV model has 89.5% of residues in favored region. This indicates both the CTL and HTL MEVs have adopted an acceptable conformation after refinement (Fig 6E and 6J) supported by the Ramachandran plot analysis that indicated acceptable conformations for both MEV models.

The designed MEVs were further tested for their potential to generate Interferon-gamma (IFN-γ) inducing epitopes required for the adaptive and the innate immune response. Therefore 15-mer peptide epitopes derived from the amino acid sequences of the designed construct of CTL and HTL MEVs were tested ability to induce IFN-γ production in CD4+T cells. A total of 33 CTL MEV and 43 HTL MEV INF-γ inducing POSITIVE epitopes with a score of 1 or more, suggesting strong potential to elicit immune response, were shortlisted (S11 Table in S1 File, Fig 6D & 6I). An IEDB database of known 3705 IFN-gamma inducing and 6728 non-inducing peptides was also utilized for prediction of INF-gamma epitopes from the constructs of CTL and HTL MEVs.

Further, structural B-cell epitopes were predicted from the final refined 3D models of CTL and HTL MEVs. The screening revealed that the CTL MEV carries 3 and the HTL MEV has 2 potential discontinuous epitopes. The score of the CTL MEV discontinuous B cell epitopes ranges from 0.682 to 0.747 and that of HTL MEV it ranges from 0.687 to 0.745 (S12 Table in S1 File, Fig 6D). As explained in method section, the higher score generated by ElliPro (closer to 0.9), indicates greatly surfaced residues and hence greater potential of the discontinuous B cell epitope.

Complementary DNA codon optimized for CTL and HTL expression in mammalian host human cell line was generated with the Java Codon Adaptation Tool. The generated optimized cDNA’s for both the MEVs were also analyzed by utilizing the GenScript Rare Codon Analysis Tool revealing a GC content of optimized CTL-MEV cDNA of 69.79% and a CAI (Codon Adaptation Index) score of 1.00 with 0% tandem rare codons. Likewise, the GC content of the optimized HTL-MEV cDNA was 70.69%, CAI score was 1.00 with 0% tandem rare codons. The CAI indicates the possibility of cDNA expression in a chosen expression system. The tandem rare codon frequency indicates the presence of low-frequency codons in the given cDNA. Since for higher possibility for cDNA expression in human expression system, the GC content of a cDNA should be within the range of 30% to 70%, the CAI score should be between 0.8–1.0, and the tandem rare codon frequency that indicates the presence of low-frequency codons, should be <30%, the cDNA constructs of CTL and HTL MPVs are expected to have high potential for expression in human expression system. The tandem rare codons may hinder the proper expression of the cDNA or even interrupt the translational machinery of the chosen expression system. According to the GenScript Rare Codon Analysis the cDNA of both the MEVs satisfy all the mentioned parameters and are predicted to have high expression in the mammalian host cell line.

#### Molecular interaction analysis of MEVs with immunological receptor

**Fig 7:**
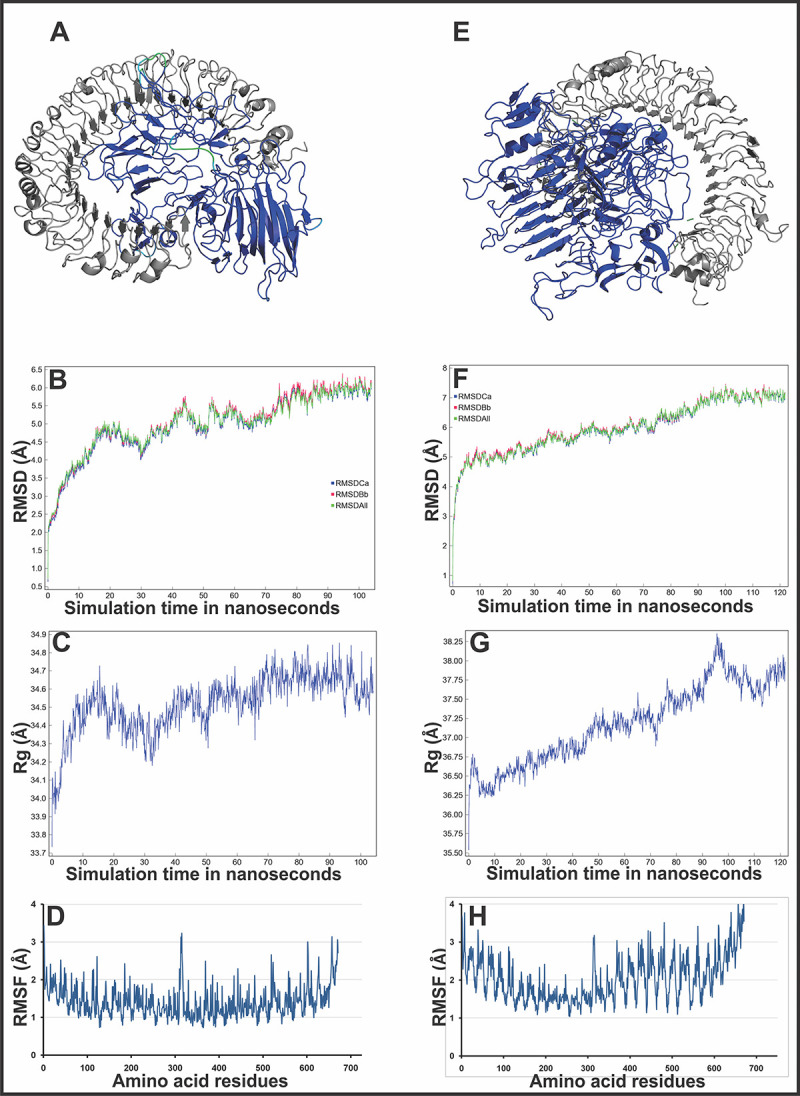
Molecular docking and dynamics simulation study of CTL and HTL MEVs with TLR3. **(A)** CTL and **(E)** HTL MEVs (VIBGYOR) docked complex with TLR3 (gray). Both the complexes are forming several hydrogen bonds in the MEV and TLR3 interface, as shown by green dots. B-Factor of the docked MEVs is shown by a rainbow (VIBGYOR) presentation. The regions in blue being indicated stable and the region in red indicate unstable. In the above complexes, most of the region of docked MEVs is in blue and with the very small region is green, yellow or orange, hence the complexes are predicted to be very stable. **(B)** and **(F),** RMSD as generated by the molecular Dynamics simulation study of CTL, HTL MEVs and TLR3 complexes. **(C)** & **(G)** Rg (radius of gyration) across the time window of 100 nanosecond. **(D)** & **(H)**, RMS fluctuation for all the atoms of the CTL, HTL MEVs and TLR3 complexes.

The refined models of CTL and HTL MEVs were further studied for their molecular interaction with the ectodomain (ECD) of human TLR3. Therefore, molecular docking of CTL and HTL MEVs models with the Human TLR3 (ECD) crystal structure model (PDB ID: 2A0Z) was performed utilizing the PatchDock tool. Generated docking conformation with highest geometric shape complementarity score of 22382 and 18264 for CTL and HTL MEVs, respectively were selected for further studies. The highest docking score predicted with the PatchDock tool indicates the best geometric shape complementarity fitting conformation of MEVs and the ectodomain of TLR3 receptor. Both, the CTL and HTL MEVs were fitting well into the ectodomain region of TLR3 after docking (Fig 7A & 7E). The CTL and HTL MEVs have shown to form multiple potential hydrogen bonds within the ectodomain cavity region of TLR3. The detailed residue interactions across interface of CTL and HTL MEVs in complex with TLR3(ECD) is generated by PDBsum and is shown in S6 Fig in S1 File. Notably the CTL MEV in complex with TLR3(ECD) forms 16 hydrogen bonds, 4 salt bridges and 850 non bonded contacts whereas HTL MEV in complex with TLR3(ECD) forms 11 hydrogen bonds, 6 salt bridges and 965 non bonded contacts. Molecular dynamics simulation study was also performed for the docked complexes of both the MEVs and TLR3 (ECD). In MD simulations both the complexes have shown reasonably stable effective RMSD (Root-mean-square deviation) value between ~ 0.2 to 4 Å for a given time window of 100 ns at invariable pressure (~ 1 bar) and temperature (~ 300 K) (Fig 7B & 7F). The effective RMSD is well within range of Hydrogen bond formation, moreover the range is towards 0.2 Å for most of duration. The reasonably invariant radius of gyration (Rg) (~ 0.7 to 2 Å) of both MEVs-TLR3 (ECD) complexes (Fig 7C & 7G), and Root-mean-square fluctuation (RMSF) (~ 2 to 3 Å) for all the atoms in both the complexes (Fig 7D & 7H) indicate that the MEVs-TLR3 complexes are stable. The B-factor analysis of MEVs-TLR3(ECD) complexes was also performed indicating the displacement of the atomic positions from an average (mean) value i.e. the more flexible an atom is the larger the displacement from the mean position will be (mean-squares displacement) (Fig 7A and 7E). The areas with high B-factors are colored red (hot), while low B-factors are colored blue (cold) (VIBGYOR presentation). The B-factor of most of the regions of MEVs-TLR3(ECD) complexes indicates the stable nature of the complexes while a very small region is found to have higher mobility. The results suggest a stable complex formation tendency for both the CTL and HTL MEVs with the ectodomain of the human TLR3 receptor.

## Conclusion

In the present study, we have designed and validated two Multi-Epitope Vaccines derived from CTL and HTL epitopes. The selected peptides show significant sequence overlap with screened linear B cell epitopes. Both, the CTL and HTL MEVs tertiary models carry potential to elicit profound humoral and cellular immune responses. Molecular interaction analysis of both MEVs in complex with immunoreceptor TLR3(ECD) showed stable structural and conformational fit of the MEVs molecule into the ectodomain of TLR3 receptor. We conclude that the CTL and HTL MEVs provided in the present study involves potential epitopes from the entire NiV proteome and cover up to 97.88% world population. The suggested MEVs provide a potential vaccine candidate against NiV for future *in vitro* and i*n vivo* validation and trial.

## Supporting information

S1 File Supporting information with supplementary figures and tables. (PDF)
