# Supplementary material for: Exploring the structural basis to develop efficient multi-epitope vaccines displaying interaction with HLA and TAP and TLR3 molecules to prevent NIPAH infection, a global threat to human health
Source: PLoS One. 2023 Mar 15;18(3):e0282580. doi: 10.1371/journal.pone.0282580 (PMC10016716; doi:10.1371/journal.pone.0282580)
Supplement: S1 File — (PDF) [file pone.0282580.s001.pdf]

Exploring the structural basis to develop efficient multi-epitope vaccines displaying interaction with HLA and TAP and TLR3 molecules to prevent NIPAH infection, a global threat to human health

Sukrit Srivastava*[1,5], Sonia Verma[2], Mohit Kamthania[1], Ajay Kumar Saxena[3], Kailash C Pandey[2], Veena Pande[4], Michael Kolbe*[5,6]

1 Infection Biology Group, Indian Foundation for Fundamental Research Trust, RaeBareli 229316, India
2 Parasite-Host Biology Group, Protein Biochemistry & Engineering Lab, ICMR-National Institute of Malaria Research, New Delhi, India
3 Molecular Medicine Lab., School of Life Science, Jawaharlal Nehru University, 110067 New Delhi, India
4 Kumaun University, Bheemtal, Nainital, Uttarakhand, India
5 Department for Structural Infection Biology, Centre for Structural Systems Biology (CSSB) & Helmholtz-Centre for Infection Research, Notkestraße 85, 22607 Hamburg, Germany.
6 Faculty of Mathematics, Informatics and Natural Sciences, University of Hamburg, Rothenbaumchaussee 19, 20148 Hamburg, Germany.

* Corresponding author
E-mail: srivastava.sukrit@iffr.in; Michael.Kolbe@helmholtz-hzi.de

S1 Table. Shortlisted high scoring CTL epitopes. Selected high scoring CTL epitopes and their respective HLA alleles binders are listed. *In-silico* analysis have shown all the selected epitopes to be non-toxic (Non-Toxin) as well as they show significant conservancy. ToxinPred analysis is based on the ToxinPred main dataset used by "ToxinPred" algorithm to predict toxicity of any unknown peptide. # Epitope match with previous studies indicating consensus in epitope screening by different approaches and methods.

#	NIPAH Proteins	Epitope	Epitopes chosen for detailed study	Position	Length	HLA Class I Alleles	Percent of protein sequence matches at 100% identity (Conservancy)	ToxinPred Study
1	C Protein	MMASILLTLF		1-10	10	B*15:01	100.00% (60/60)	Non-Toxin
2	Fusion Protein	AQITAGVALY		126-135	10	B*15:01	97.30% (36/37)	Non-Toxin
3	Fusion Protein	KYLSDLLFVF	√	205-214	10	A*23:01<br>A*24:02	97.30% (36/37)	Non-Toxin
4	Fusion Protein	MTIQAISQAF		226-235	10	B*15:01	97.30% (36/37)	Non-Toxin
5	Fusion Protein	FALSNGVLF#		376-384	9	B*35:01	100.00% (37/37)	Non-Toxin
6	Glycoprotein	LAMDEGYFAY		222-231	10	B*35:01	90.91% (60/66)	Non-Toxin
7	Glycoprotein	TVYHCSAVY#		278-286	9	A*30:02	100.00% (66/66)	Non-Toxin
8	Glycoprotein	AVYNNEFYY	√	284-292	9	A*11:01<br>A*30:02	90.91% (60/66)	Non-Toxin
9	Glycoprotein	AENPVFTVF	√	532-540	9	B*44:03<br>B*44:02	100.00% (66/66)	Non-Toxin
10	Matrix Protein	NYMYLICYGF		61-70	10	A*23:01	97.44% (38/39)	Non-Toxin
11	Matrix Protein	YMIPRTMLEF		187-196	10	B*15:01	97.44% (38/39)	Non-Toxin
12	Nucleocapsid	TPFVDSRAY		143-151	9	B*35:01	100.00% (13/13)	Non-Toxin
13	Nucleocapsid	EIISDIGNY		250-258	9	A*26:01	100.00% (13/13)	Non-Toxin
14	Nucleocapsid	YPALALNEF#		279-287	9	B*35:01	100.00% (13/13)	Non-Toxin
15	Phosphoprotein/V Protein/ W protein	LDPVVTDVVY	√	107-116	10	B*35:01	100.00% (27/27)	Non-Toxin
16	Phosphoprotein/V Protein/ W protein	LVSDAKMLSY		150-159	10	A*01:01	59.26% (16/27)	Non-Toxin
17	Phosphoprotein/V Protein/ W protein	VSDAKMLSY#	√	151-159	9	A*01:01	59.26% (16/27)	Non-Toxin
18	Phosphoprotein	MPSDDFSNTF		479-488	10	B*35:01	96.30% (26/27)	Non-Toxin
19	Polymerase	YPECNNILF#		88-96	9	B*35:01	90.00% (9/10)	Non-Toxin
20	Polymerase	IMKKSFKAY		110-118	9	B*15:01	100.00% (10/10)	Non-Toxin
21	Polymerase	KWYECFLFWF		165-174	10	A*23:01	100.00% (10/10)	Non-Toxin
22	Polymerase	FPVMGNRIY#		276-284	9	B*35:01	100.00% (10/10)	Non-Toxin
23	Polymerase	AEFFSFFRTF#	√	354-363	10	B*44:03	100.00% (10/10)	Non-Toxin
24	Polymerase	IPFLFLSAY#		811-819	9	B*35:01	100.00% (10/10)	Non-Toxin
25	Polymerase	IATVYTWAY		1300-1308	9	B*35:01	100.00% (10/10)	Non-Toxin
26	Polymerase	LETDDYNGIY		1411-1420	10	A*01:01	100.00% (10/10)	Non-Toxin
27	Polymerase	ETDDYNGIY#		1412-1420	9	A*01:01	100.00% (10/10)	Non-Toxin
28	Polymerase	SQNLLVTSY#		1624-1632	9	B*15:01	100.00% (10/10)	Non-Toxin
29	Polymerase	TSDLDFVIFY		1716-1725	10	A*01:01	100.00% (10/10)	Non-Toxin
30	Polymerase	FPISRLFNMY		1983-1992	10	B*35:01	100.00% (10/10)	Non-Toxin
31	Polymerase	RLFNMYRSY	√	1987-1995	9	A*32:01<br>B*15:01 A*30:02	100.00% (10/10)	Non-Toxin
32	Polymerase	SYFGLVLVCF		1994-2003	10	A*23:01	100.00% (10/10)	Non-Toxin
33	Polymerase	KYYQIDQPFF		2068-2077	10	A*23:01	100.00% (10/10)	Non-Toxin

S2 Table. CTL epitope prediction. Detailed scoring of all screened CTL epitopes and their respective HLA class I allele binders. CTL epitopes were chosen on the basis of high "Total score" and higher number of HLA allele binders. Total score is a combined score of TAP score, MHC score, Proteasome score and Processing score.

#	NIPAH-CoV Protein	Epitope	HLA Class I Alleles	Proteasome Score	TAP Score	MHC Score	Processing Score	Total Score	MHC IC50[nM]	Immunogenicity-Score
1	C Protein	MMASILLTLF	B*15:01	1.2	1.13	-0.76	2.33	1.57	5.8	-0.01913
2	Fusion Protein	AQITAGVALY	B*15:01	1.18	1.46	-1	2.64	1.64	10	0.21876
3	Fusion Protein	KYLSDLLFVF	A*23:01	1.55	1.29	-0.74	2.84	2.1	5.5	-0.04679
	Fusion Protein		A*24:02	1.55	1.29	-0.98	2.84	1.86	9.5	-0.04679
4	Fusion Protein	MTIQAISQAF	B*15:01	1.4	1.16	-0.92	2.57	1.64	8.4	-0.13629
5	Fusion Protein	FALSNGVLF	B*35:01	1.49	1.15	-0.77	2.64	1.86	5.9	-0.11611
6	Glycoprotein	LAMDEGYFAY	B*35:01	1.43	1.34	-0.85	2.77	1.93	7	0.214
7	Glycoprotein	TVYHCSAVY	A*30:02	1.57	1.46	-1.51	3.03	1.52	32.6	-0.11974
8	Glycoprotein	AVYNNEFYY	A*11:01	1.21	1.5	-1.07	2.7	1.63	11.8	0.17688
	Glycoprotein		A*30:02	1.21	1.5	-1.11	2.7	1.59	13	0.17688
9	Glycoprotein	AENPVFTVF	B*44:03	1.71	1.07	-1.09	2.77	1.69	12.3	0.19402
	Glycoprotein		B*44:02	1.71	1.07	-1.21	2.77	1.56	16.3	0.19402
10	Matrix Protein	NYMYLICYGF	A*23:01	1.29	1.32	-1.09	2.61	1.52	12.2	0.02401
11	Matrix Protein	YMIPRTMLEF	B*15:01	1.4	1.18	-0.79	2.57	1.79	6.1	0.00408
12	Nucleocapsid	TPFVDSRAY	B*35:01	1.33	1.22	-0.79	2.56	1.77	6.1	0.01195
13	Nucleocapsid	EIISDIGNY	A*26:01	1.19	1.29	-0.6	2.47	1.87	4	0.04843
14	Nucleocapsid	YPALALNEF	B*35:01	1.25	1.02	-0.61	2.27	1.66	4.1	0.08224
15	Phosphoprotein/V Protein/ W protein	LDPVVTDVVY	B*35:01	1.74	1.11	-1.34	2.85	1.51	22.1	0.19578
16	Phosphoprotein/V Protein/ W protein	LVSDAKMLSY	A*01:01	1.48	1.33	-1.26	2.81	1.55	18	-0.4746
17	Phosphoprotein/V Protein/ W protein	VSDAKMLSY	A*01:01	1.48	1.26	-0.94	2.74	1.8	8.7	-0.43475
18	Phosphoprotein	MPSDDFSNTF	B*35:01	1.53	0.99	-0.88	2.52	1.64	7.6	-0.03429
19	Polymerase	YPECNNILF	B*35:01	1.47	0.96	-0.82	2.44	1.62	6.6	0.0717
20	Polymerase	IMKKSFKAY	B*15:01	1.59	1.39	-1.07	2.98	1.92	11.7	-0.49704
21	Polymerase	KWYECFLFWF	A*23:01	1.24	1.36	-1	2.6	1.6	10.1	0.37883
22	Polymerase	FPVMGNRIY	B*35:01	1.57	1.15	-0.51	2.72	2.22	3.2	-0.01495
13	Polymerase	AEFFSFFRTF	B*44:03	1.49	1.17	-1.05	2.66	1.61	11.3	0.28526
24	Polymerase	IPFLFLSAY	B*35:01	1.39	1.2	-0.67	2.59	1.92	4.7	0.01364
25	Polymerase	IATVYTWAY	B*35:01	1.56	1.34	-0.93	2.9	1.97	8.6	0.29688
26	Polymerase	LETDDYNGIY	A*01:01	1.41	1.25	-0.93	2.66	1.73	8.6	0.15319
27	Polymerase	ETDDYNGIY	A*01:01	1.41	1.12	-0.91	2.53	1.62	8.1	0.12619
28	Polymerase	SQNLLVTSY	B*15:01	1.6	1.31	-1.38	2.9	1.52	24.2	-0.0491
29	Polymerase	TSDLDFVIFY	A*01:01	1.68	1.26	-0.87	2.94	2.07	7.4	0.35122
30	Polymerase	FPISRLFNMY	B*35:01	1.46	1.11	-0.98	2.57	1.59	9.6	-0.08153
31	Polymerase	RLFNMYRSY	A*32:01	1.42	1.46	-1.11	2.88	1.77	12.8	-0.19597
	Polymerase		B*15:01	1.42	1.46	-1.18	2.88	1.69	15.3	-0.19597
	Polymerase		A*30:02	1.42	1.46	-1.2	2.88	1.68	15.8	-0.19597
32	Polymerase	SYFGLVLVCF	A*23:01	1.48	1.34	-1.04	2.82	1.78	10.9	0.0944
33	Polymerase	KYYQIDQPFF	A*23:01	1.33	1.39	-0.98	2.72	1.73	9.6	-0.01656

S3 Table. Shortlisted high scoring HTL epitopes. Selected high scoring HTL epitopes and their respective HLA alleles binders are listed above. *In-silico* analysis have shown all the selected epitopes to be non-toxic (Non-Toxin) as well as they show significant conservancy.

#	NIPAH Protein	Epitope	Epitopes chosen for detailed study	Position	Length	HLA Class II Alleles	Percent of protein sequence matches at 100% identity (Conservancy)	ToxinPred Study
1	C Protein	VQMTYNWTQWLQTLY		103-117	15	DPA1*01:03 DPB1*02:01	95.00% (57/60)	Non-Toxin
2	Fusion protein	DLALSKYLSDLLFVF	√	104-118	15	DRB3*01:01	97.30% (36/37)	Non-Toxin
3	Fusion protein	LALSKYLSDLLFVFG	√	59-73	15	DRB3*01:01	97.30% (36/37)	Non-Toxin
4	Fusion protein	ALSKYLSDLLFVFGP	√	99-113	15	DRB3*01:01	97.30% (36/37)	Non-Toxin
5	Fusion protein	LSKYLSDLLFVFGPN	√	100-114	15	DRB3*01:01	97.30% (36/37)	Non-Toxin
6	Fusion protein	SKYLSDLLFVFGPNL	√	101-115	15	DRB3*01:01	97.30% (36/37)	Non-Toxin
7	Fusion protein	KYLSDLLFVFGPNLQ	√	102-116	15	DRB3*01:01	97.30% (36/37)	Non-Toxin
8	Fusion protein	YLSDLLFVFGPNLQD	√	103-117	15	DRB3*01:01	97.30% (36/37)	Non-Toxin
9	Glycoprotein	ASFSWDTMIKFGDVL		456-470	15	DRB3*01:01	19.70% (13/66)	Non-Toxin
10	Glycoprotein	FSWDTMIKFGDVLTV		458-472	15	DRB3*01:01	19.70% (13/66)	Non-Toxin
11	Glycoprotein	GVYNDAFLIDRINWI		506-520	15	DRB3*01:01	100.00% (66/66)	Non-Toxin
12	Glycoprotein	NDAFLIDRINWISAG		509-523	15	DRB3*01:01	100.00% (66/66)	Non-Toxin
13	Glycoprotein	DAFLIDRINWISAGV		510-424	15	DRB3*01:01	100.00% (66/66)	Non-Toxin
14	Glycoprotein	AFLIDRINWISAGVF		511-525	15	DRB3*01:01	100.00% (66/66)	Non-Toxin
15	Glycoprotein	FLIDRINWISAGVFL		512-526	15	DRB3*01:01	100.00% (66/66)	Non-Toxin
16	Matrix Protein	IPREFMIYDDVFIDN		331-345	15	DRB3*01:01	38.46% (15/39)	Non-Toxin
17	Matrix Protein	FMIYDDVFIDNTGRI		335-349	15	DRB3*01:01	94.87% (37/39)	Non-Toxin
18	Nucleocapsid	LSSDQVAELAAAVQE		384-398	15	DQA1*04:01 DQB1*04:02	47.37% (9/19)	Non-Toxin
19	Nucleocapsid	SSDQVAELAAAVQET		385-399	15	DQA1*04:01 DQB1*04:02	47.37% (9/19)	Non-Toxin
20	Nucleocapsid	SDQVAELAAAVQETS		386-400	15	DQA1*04:01 DQB1*04:02	47.37% (9/19)	Non-Toxin
21	Nucleocapsid	DQVAELAAAVQETSA		387-401	15	DQA1*04:01 DQB1*04:02	47.37% (9/19)	Non-Toxin
22	Nucleocapsid	QVAELAAAVQETSAG		388-402	15	DQA1*04:01 DQB1*04:02	100.00% (19/19)	Non-Toxin
23	Phosphoprotein/V Protein/ W protein	NNGNVCLVSDAKMLS	√	144-158	15	DRB1*03:01	62.96% (17/27)	Non-Toxin
24	Phosphoprotein/V Protein/ W protein	NGNVCLVSDAKMLSY	√	145-159	15	DRB1*03:01	59.26% (16/27)	Non-Toxin
25	Phosphoprotein/V Protein/ W protein	GNVCLVSDAKMLSYA	√	146-160	15	DRB1*03:01	59.26% (16/27)	Non-Toxin
26	Phosphoprotein/V Protein/ W protein	NVCLVSDAKMLSYAP	√	147-161	15	DRB1*03:01	59.26% (16/27)	Non-Toxin
27	Phosphoprotein/V Protein/ W protein	VCLVSDAKMLSYAPE	√	148-162	15	DRB1*03:01	59.26% (16/27)	Non-Toxin
28	Polymerase	NIDNIHLLAEFFSFF	√	346-360	15	DPA1*01:03 DPB1*02:01	100.00% (10/10)	Non-Toxin
29	Polymerase	IDNIHLLAEFFSFFR	√	347-361	15	DPA1*01:03 DPB1*02:01	100.00% (10/10)	Non-Toxin
30	Polymerase	DNIHLLAEFFSFFRT	√	348-362	15	DPA1*01:03 DPB1*02:01	100.00% (10/10)	Non-Toxin
31	Polymerase	NIHLLAEFFSFFRTF	√	349-363	15	DPA1*01:03 DPB1*02:01	100.00% (10/10)	Non-Toxin
32	Polymerase	IHLLAEFFSFFRTFG	√	350-364	15	DPA1*01:03 DPB1*02:01	100.00% (10/10)	Non-Toxin
33	Polymerase	LELASFLMDRRVILP		1102-1116	15	DRB3*01:01	100.00% (10/10)	Non-Toxin
34	Polymerase	ELASFLMDRRVILPR		1103-1117	15	DRB3*01:01	100.00% (10/10)	Non-Toxin
35	Polymerase	LASFLMDRRVILPRA		1104-1118	15	DRB3*01:01	100.00% (10/10)	Non-Toxin
36	Polymerase	ASFLMDRRVILPRAA		1105-1119	15	DRB3*01:01	100.00% (10/10)	Non-Toxin
37	Polymerase	LDFVIFYASLTYLRR		1719-1733	15	DPA1*02:01 DPB1*14:01	100.00% (10/10)	Non-Toxin
38	Polymerase	FVIFYASLTYLRRGI		1721-1735	15	DPA1*02:01 DPB1*14:01	100.00% (10/10)	Non-Toxin

S4 Table. HTL epitope prediction. Percentile rank of HTL epitopes and their respective HLA class II allele binders. HTL epitopes were screened on the basis of percentile rank (lower the percentile number, higher the rank) and larger number of HLA allele binders. Last column show the method used for epitope screening.

#	Epitope	Epitope	HLA Class II Alleles	Percentile rank	Method used
1	C Protein	VQMTYNWTQWLQTLY	DPA1*01:03 DPB1*02:01	0.08	Consensus (comb.lib./smm/nn)
2	Fusion Protein	DLALSKYLSDLLFVF	DRB3*01:01	0.01	Consensus (comb.lib./smm/nn)
3	Fusion Protein	LALSKYLSDLLFVFG	DRB3*01:01	0.01	Consensus (comb.lib./smm/nn)
4	Fusion Protein	ALSKYLSDLLFVFGP	DRB3*01:01	0.01	Consensus (comb.lib./smm/nn)
5	Fusion Protein	LSKYLSDLLFVFGPN	DRB3*01:01	0.01	Consensus (comb.lib./smm/nn)
6	Fusion Protein	SKYLSDLLFVFGPNL	DRB3*01:01	0.01	Consensus (comb.lib./smm/nn)
7	Fusion Protein	KYLSDLLFVFGPNLQ	DRB3*01:01	0.01	Consensus (comb.lib./smm/nn)
8	Fusion Protein	YLSDLLFVFGPNLQD	DRB3*01:01	0.01	Consensus (comb.lib./smm/nn)
9	Glycoprotein	ASFSWDTMIKFGDVL	DRB3*01:01	0.01	Consensus (comb.lib./smm/nn)
10	Glycoprotein	FSWDTMIKFGDVLTV	DRB3*01:01	0.01	Consensus (comb.lib./smm/nn)
11	Glycoprotein	GVYNDAFLIDRINWI	DRB3*01:01	0.01	Consensus (comb.lib./smm/nn)
12	Glycoprotein	NDAFLIDRINWISAG	DRB3*01:01	0.01	Consensus (comb.lib./smm/nn)
13	Glycoprotein	DAFLIDRINWISAGV	DRB3*01:01	0.01	Consensus (comb.lib./smm/nn)
14	Glycoprotein	AFLIDRINWISAGVF	DRB3*01:01	0.01	Consensus (comb.lib./smm/nn)
15	Glycoprotein	FLIDRINWISAGVFL	DRB3*01:01	0.01	Consensus (comb.lib./smm/nn)
16	Matrix Protein	IPREFMIYDDVFIDN	DRB3*01:01	0.01	Consensus (comb.lib./smm/nn)
17	Matrix Protein	FMIYDDVFIDNTGRI	DRB3*01:01	0.01	Consensus (comb.lib./smm/nn)
18	Nucleocapsid	LSSDQVAELAAAVQE	DQA1*04:01 DQB1*04:02	0.01	Consensus (comb.lib./smm/nn)
19	Nucleocapsid	SSDQVAELAAAVQET	DQA1*04:01 DQB1*04:02	0.01	Consensus (comb.lib./smm/nn)
20	Nucleocapsid	SDQVAELAAAVQETS	DQA1*04:01 DQB1*04:02	0.01	Consensus (comb.lib./smm/nn)
21	Nucleocapsid	DQVAELAAAVQETSA	DQA1*04:01 DQB1*04:02	0.01	Consensus (comb.lib./smm/nn)
22	Nucleocapsid	QVAELAAAVQETSAG	DQA1*04:01 DQB1*04:02	0.01	Consensus (comb.lib./smm/nn)
23	Phosphoprotein/V Protein/ W protein	NNGNVCLVSDAKMLS	DRB1*03:01	0.01	Consensus (comb.lib./smm/nn)
24	Phosphoprotein/V Protein/ W protein	NGNVCLVSDAKMLSY	DRB1*03:01	0.01	Consensus (comb.lib./smm/nn)
25	Phosphoprotein/V Protein/ W protein	GNVCLVSDAKMLSYA	DRB1*03:01	0.01	Consensus (comb.lib./smm/nn)
26	Phosphoprotein/V Protein/ W protein	NVCLVSDAKMLSYAP	DRB1*03:01	0.01	Consensus (comb.lib./smm/nn)
27	Phosphoprotein/V Protein/ W protein	VCLVSDAKMLSYAPE	DRB1*03:01	0.01	Consensus (comb.lib./smm/nn)
28	Polymerase	NIDNIHLLAEFFSFF	DPA1*01:03 DPB1*02:01	0.01	Consensus (comb.lib./smm/nn)
29	Polymerase	IDNIHLLAEFFSFFR	DPA1*01:03 DPB1*02:01	0.01	Consensus (comb.lib./smm/nn)
30	Polymerase	DNIHLLAEFFSFFRT	DPA1*01:03 DPB1*02:01	0.01	Consensus (comb.lib./smm/nn)
31	Polymerase	NIHLLAEFFSFFRTF	DPA1*01:03 DPB1*02:01	0.01	Consensus (comb.lib./smm/nn)
32	Polymerase	IHLLAEFFSFFRTFG	DPA1*01:03 DPB1*02:01	0.01	Consensus (comb.lib./smm/nn)
33	Polymerase	LELASFLMDRRVILP	DRB3*01:01	0.01	Consensus (comb.lib./smm/nn)
34	Polymerase	ELASFLMDRRVILPR	DRB3*01:01	0.01	Consensus (comb.lib./smm/nn)
35	Polymerase	LASFLMDRRVILPRA	DRB3*01:01	0.01	Consensus (comb.lib./smm/nn)
36	Polymerase	ASFLMDRRVILPRAA	DRB3*01:01	0.01	Consensus (comb.lib./smm/nn)
37	Polymerase	LDFVIFYASLTYLRR	DPA1*02:01 DPB1*14:01	0.01	Consensus (comb.lib./smm/nn)
38	Polymerase	FVIFYASLTYLRRGI	DPA1*02:01 DPB1*14:01	0.01	Consensus (comb.lib./smm/nn)

S5 Table. Shortlisted B Cell epitopes. BepiPred Linear B Cell epitopes showing sequence overlap with CTL and HTL epitopes are shortlisted above. *In-silico* analysis have shown all the selected epitopes to be non-toxic (Non-Toxin) as well as they show significant amino acid sequence conservancy. #Epitope match with previous studies indicating consensus in epitope screening by different approaches and methods.

#	NIPAH Protein	B Cell Epitope	Position	Length	Percent of protein sequence matches at 100% identity (Conservancy)	ToxinPred Study
1	Fusion Protein	GPNLQDPVSNSM	215-226	12	97.30% (36/37)	Non-Toxin
2	Glycoprotein	WTPPNPNT	271-278	8	22.73% (15/66)	Non-Toxin
3	Glycoprotein	SWDTMI	459-464	6	100.00% (66/66)	Non-Toxin
4	Glycoprotein	NQTAE	529-533	5	100.00% (66/66)	Non-Toxin
5	Matrix Protein	SGIYM	184-188	5	97.44% (38/39)	Non-Toxin
6	Matrix Protein	SIPREFMIY#	330-338	9	38.46% (15/39)	Non-Toxin
7	Matrix Protein	DVFIDNTGRI	340-349	10	94.87% (37/39)	Non-Toxin
8	Phosphoprotein	GYGFTSSPERGWSDYTSGA	125-143	19	51.85% (14/27)	Non-Toxin
9	Phosphoprotein	IAVSKEDR	163-170	8	100.00% (27/27)	Non-Toxin
10	Polymerase	NIDN	346-349	4	100.00% (10/10)	Non-Toxin
11	Polymerase	GHPILE	364-369	6	100.00% (10/10)	Non-Toxin
12	Polymerase	DKSFDEDLEL	1095-1104	10	100.00% (10/10)	Non-Toxin
13	Polymerase	LMDR	1108-1111	4	100.00% (10/10)	Non-Toxin
14	Polymerase	LRLETDDYNG	1409-1418	10	100.00% (10/10)	Non-Toxin
15	Polymerase	GFPIS	1982-1986	5	100.00% (10/10)	Non-Toxin
16	Polymerase	PVYSNPD	2004-2010	7	100.00% (10/10)	Non-Toxin

S6 Table. Protein sequence retrieval, tertiary structures retrieval and homology modeling of nine Nipah proteins. Nipah protein sequences were retrieved from NCBI. Available structure files (pdb) for Nipah proteins were retrieved from RCSB PDB. Nipah proteins with no tertiary structure available were subjected to homology modeling by Swissmodel.

#	NIPAH-CoV Protein	Number of sequence retrieved from NCBI/PDB	PDB ID of available structure	Template used for modeling	QMEAN
1	C Protein	13	-	5hyb.1.B	- 2.27
2	Fusion Protein	18	5EVM	-	-
3	Glycoprotein	21	2VWD	-	-
4	Matrix Protein	18	-	6bk6.1.A	- 1.81
5	Nucleocapsid	36	4CO6	-	-
6	Phosphoprotein	18	4N5B	-	-
7	Polymerase	18	-	5a22.1.A	-6.06
8	V protein	11	-	4co6.1.B	-0.11
9	W protein	08	6BW0	-	-

S7 Table. World population coverage by the shortlisted CTL and HTL epitopes combined. The cumulative percent of world (countries as listed in table) population coverage is 97.88% by the joint administration of selected CTL and HTL epitopes as vaccine candidate.

a projected population coverage
b average number of epitope hits / HLA combinations recognized by the population
c minimum number of epitope hits / HLA combinations recognized by 90% of the population

Population/Area	Class combined		
	coverage[a]	average_hit[b]	pc90[c]
Austria	86.74%	7.22	0.75
Belgium	82.97%	5.68	0.59
Borneo	9.26%	0.52	0.55
Brazil	99.93%	13.73	7.16
Canada	27.97%	1.53	0.69
Central Africa	95.73%	10.45	2.84
Central America	50.90%	2.48	0.41
Chile	81.43%	4.35	0.54
China	96.24%	8.75	2
Cuba	81.77%	5.29	0.55
Czech Republic	82.95%	7.12	0.59
Denmark	27.87%	1.51	0.69
East Africa	95.36%	10.37	2.87
East Asia	93.70%	7.99	1.29
England	90.31%	7.45	1.03
Europe	99.91%	13.69	6.97
Finland	85.91%	8.29	0.71
France	99.98%	13.86	7.26
Georgia	88.89%	7.81	0.9
Germany	89.61%	7.97	0.96
Hong Kong	75.06%	1.63	0.4
India	99.13%	10.73	6.15
Indonesia	70.81%	2.13	0.34
Iran	74.68%	4.17	0.39
Ireland South	91.40%	7.24	1.14
Israel	72.73%	3.85	0.37
Italy	95.31%	10.89	2.76
Japan	98.13%	11.21	3.28
Jordan	55.10%	1.58	0.22
Kenya	94.26%	9.77	2.59
Korea; South	86.85%	5.82	0.76
Lebanon	54.37%	3.41	0.44
Malaysia	66.85%	2.91	0.3
Mexico	99.99%	13.79	6.69
Mongolia	87.73%	5.75	0.82
Netherlands	45.52%	2.86	0.37
North Africa	85.76%	7.63	0.7
North America	99.99%	13.61	6.86
Northeast Asia	96.28%	8.71	1.98
Norway	49.89%	3.23	0.4
Oceania	97.70%	9.04	2.13
Oman	81.20%	3.52	0.53
Pakistan	71.59%	1.54	0.35
Peru	83.62%	6.66	0.61
Philippines	63.66%	0.79	0.28

Population/Area	Class combined		
	coverage[a]	average_hit[b]	pc90[c]
Poland	83.36%	5.59	0.6
Portugal	81.92%	5.06	0.55
Romania	80.31%	4.61	0.51
Russia	99.80%	14.12	7.27
Saudi Arabia	73.55%	4.44	0.38
Scotland	55.20%	1.76	0.22
Singapore	75.29%	2.61	0.4
Slovakia	45.98%	2.94	0.37
South Africa	75.52%	4.38	0.41
South America	97.92%	10.72	4.7
South Asia	99.33%	10.98	6.22
Southeast Asia	82.34%	3.19	0.57
Southwest Asia	79.01%	5.18	0.48
Spain	99.93%	10.55	6.29
Sri Lanka	38.06%	1.23	0.16
Sri Lanka Asian	38.06%	1.23	0.16
Sweden	99.96%	15.31	7.31
Taiwan	90.12%	4.26	1
Thailand	78.15%	3.4	0.46
Turkey	23.60%	1.25	0.65
United Arab Emirates	33.67%	1.73	0.71
United Kingdom	46.56%	1.86	0.37
United States	100.00%	13.38	6.81
Vietnam	75.66%	2.48	0.41
West Africa	98.14%	11.01	2.78
West Indies	85.36%	6.22	0.68
Zimbabwe	82.41%	6.54	0.57
**Cumulative percent of world population coverage**	**97.88%**	**11.33**	**4.99**
**Average population coverage**	**77.84**	**6.38**	**1.87**
**Standard deviation**	**21.97**	**4.07**	**2.3**

S8 Table. Homology modeling for HLA alleles. Tertiary structure of HLA alleles were modeled by homology modeling using SwissModel server. Templates were chosen with highest sequence identity. Generated models with acceptable QMEAN value were chosen for further studies.

#	HLA class I allele	Template used for modeling (PDB IDs)	% sequence identity	QMEAN
1	A0101	4nqx.2.A	100%	(-)0.07
2	A3002	6eny.1.D	96.48%	(-)0.52
3	A2301	2bck.1.A	98.91%	(-)0.01
4	A2402	2bck.1.A	100%	(-)0.02
5	B1501	5txs.1.A	100%	0.14
6	B3501	1a9b.1.A	100%	(-)0.60
7	A1101	6eny.1.D	97.95%	0.75
8	A3201	6ei2.1.A	92.00%	0.57
9	B4402	1m6o.1.A	100%	0.75
10	B4403	4jqx.1.A	100%	0.64
#	HLA class II allele	Template used for modeling (PDB IDs)	% sequence identity	QMEAN
2	DPA1-0103	4p4r.1.A	100%	(-)0.73
2	DRB3-0101	2q6w.1.B	100%	(-)0.65
6	DPB1-0201	4p5m.1.B	98.42%	(-)0.37
4	DRB1-0301	1a6a.1.B	100%	(-)1.65

S9 Table. Prevalence of amino acids of epitopes binding to the HLA allele molecules. The count of times residue is involved in interaction is shown in last column.

CTL Epitopes residues involved in interaction with class I HLA alleles

AEFF SFFR TF-B4403	AENP VFTV F-B4402	AENP VFTV F-B4403	AVYN NEFY Y-A1101	AVYN NEFY Y-A3002	KYLS DLLF VF-A2301	KYLS DLLF VF-A2402	LDPV VTDV VY-B3501	RLFN MYRS Y-A3002	RLFN MYRS Y-A3201	RLFN MYRS Y-B1501	VSDA KMLS Y-A0101	CTL epitope Res
F	F	F	F		F, F				F			**7**
			Y, Y	Y, Y	Y			Y, Y	Y	Y, Y		**10**
S					S			S	S	S	S, S	**7**
	N	N		N, N				N		N		**6**
							D					**1**
E	E	E	E									**4**
						L				L		**2**
R								R	R	R, R		**5**
		V	V				V					**3**
						K						**1**
		T										**1**

CTL & HTL Epitopes residues involved in interaction with class I & II HLA alleles respectively
(The count of times residue is involved in interaction is shown next to the residues)

F-23; Y-17; S-17; N-16; D-13; E-11; L-11
R-10; A-6; V-5; K-5; H-5; G-5; M-3; P-2
C-2; I-2; T-1; Q-1

HTL Epitopes residues involved in interaction with class II HLA alleles

ALSK YLSD LLFVF GP-DRB3-0101	DLAL SKYL SDLL FVF-DRB3-0101	DNIHL LAEF FSFF RT-DPA1-0103	DNIHL LAEF FSFF RT-DPB1-0201	GNVC LVSD AKML SYA-DRB1-0301	IDNIH LLAE FFSF FR-DPA1-0103	IDNIH LLAE FFSF FR-DPB1-0201	IHLLA EFFS FFRT FG-DPA1-0103	IHLLA EFFS FFRT FG-DPB1-0201	KYLS DLLFV FGPN LQ-DRB3-0101	LALSK YLSD LLFVF G-DRB3-0101	LSKYL SDLLF VFGP N-DRB3-0101	NGNV CLVS DAKM LSY-DRB1-0301	NIDNI HLLA EFFS FF-DPA1-0103	NIDNI HLLA EFFS FF-DPB1-0201	NIHLL AEFF SFFR TF-DPA1-0103	NIHLLAE FFSFFRT F-DPB1-0201	NNGNV CLVSDA KMLS-DRB1-0301	NVC LVS DAK MLS YAP-DRB 1-0301	SKY LSD LLF VFG PNL-DRB 3-0101	VCL VSD AKM LSY APE-DRB 1-0301	YLSD LLFV FGPN LQD-DRB3 -0101	HTL epitope Res
F	F,F	F			F	F				F	F		F, F	F, F		F, F, F, F						**16**
Y				Y							Y	Y						Y	Y	Y		**7**
	S					S		S	S	S, S	S								S	S	S	**10**
				N							N	N, N		N		N	N, N, N	N				**10**
D, D	D,D	D	D								D		D	D				D	D	D		**12**
			E			E	E	E					E	E						E		**7**
L	L		L	L, L					L	L				L				L				**9**
			R		R		R	R								R						**5**
		A			A							A			A			A		A		**6**
				V														V				**2**
									K								K	K	K			**4**
					H	H	H						H		H							**5**
								G		G	G	G					G					**5**
																	M	M		M		**3**
P											P											**2**
				C																C		**2**
							I							I								**2**
									Q													**1**

S10 Table. Refinement models of CTL and HTL MEVs. CTL and HTL MEVs models were refined by GalaxyWEB server and used for further studies. After refinement in particular Rama favored residues increased significantly.

Galaxy Refinement for CTL MEV						
Model	GDT-HA	RMSD	MolProbity	Clash score	Poor rotamers	Rama favored
Initial	1.00	0.00	3.336	120.1	1.8	87.6
MODEL 1	0.9596	0.385	2.673	29.9	1.8	90.8
Galaxy Refinement for HTL MEV						
Initial	1.00	0.00	3.725	167.4	3.4	86.2
MODEL 1	0.9463	0.419	2.811	38.4	1.6	88.5

S11 Table. INF-γ epitopes from CTL and HTL MEVs. INF-γ inducing (POSITIVE) epitopes from CTL and HTL MEVs were screened by using "Motif and SVM hybrid" (MERCI & SVM) approaches.

CLT Epitopes also predicted to be IFN-gamma epitopes					
#	Start-END	Sequence	Method	Result	Score
1	22-37	RRYKQIGTCGLPGTK	MERCI	POSITIVE	1
2	23-38	RYKQIGTCGLPGTKC	MERCI	POSITIVE	1
3	24-39	YKQIGTCGLPGTKCC	MERCI	POSITIVE	1
4	25-40	KQIGTCGLPGTKCCK	MERCI	POSITIVE	1
5	26-41	QIGTCGLPGTKCCKK	MERCI	POSITIVE	1
6	38-53	CKKPEAAAKMMASIL	MERCI	POSITIVE	1
7	112-127	GVLFGGGGSLAMDEG	MERCI	POSITIVE	1
8	113-128	VLFGGGGSLAMDEGY	MERCI	POSITIVE	1
9	114-129	LFGGGGSLAMDEGYF	MERCI	POSITIVE	1
10	115-130	FGGGGSLAMDEGYFA	MERCI	POSITIVE	1
11	116-131	GGGGSLAMDEGYFAY	MERCI	POSITIVE	1
12	117-132	GGGSLAMDEGYFAYG	MERCI	POSITIVE	1
13	118-133	GGSLAMDEGYFAYGG	MERCI	POSITIVE	1
14	119-134	GSLAMDEGYFAYGGG	MERCI	POSITIVE	1
15	120-135	SLAMDEGYFAYGGGG	MERCI	POSITIVE	1
16	325-340	KKSFKAYGGGGSKWY	MERCI	POSITIVE	1
17	326-341	KSFKAYGGGGSKWYE	MERCI	POSITIVE	1
18	327-342	SFKAYGGGGSKWYEC	MERCI	POSITIVE	1
19	328-343	FKAYGGGGSKWYECF	MERCI	POSITIVE	1
20	329-344	KAYGGGGSKWYECFL	MERCI	POSITIVE	2
21	330-345	AYGGGGSKWYECFLF	MERCI	POSITIVE	2
22	331-346	YGGGGSKWYECFLFW	MERCI	POSITIVE	2
23	332-347	GGGGSKWYECFLFWF	MERCI	POSITIVE	2
24	333-348	GGGSKWYECFLFWFG	MERCI	POSITIVE	1
25	334-349	GGSKWYECFLFWFGG	MERCI	POSITIVE	1
26	335-350	GSKWYECFLFWFGGG	MERCI	POSITIVE	1
27	336-351	SKWYECFLFWFGGGG	MERCI	POSITIVE	1
28	337-352	KWYECFLFWFGGGGS	MERCI	POSITIVE	1
29	388-403	AYGGGGSIATVYTWA	MERCI	POSITIVE	1
30	392-407	GGSIATVYTWAYGGG	MERCI	POSITIVE	1
31	393-408	GSIATVYTWAYGGGG	MERCI	POSITIVE	1
32	394-409	SIATVYTWAYGGGGS	MERCI	POSITIVE	1
33	559-572	STRGRKCCRRKKHHH	MERCI	POSITIVE	1
HLT Epitopes also predicted to be IFN-gamma epitopes					
#	Start-END	Sequence	Method	Result	Score
1	22-37	RRYKQIGTCGLPGTK	MERCI	POSITIVE	1
2	23-38	RYKQIGTCGLPGTKC	MERCI	POSITIVE	1
3	24-39	YKQIGTCGLPGTKCC	MERCI	POSITIVE	1
4	25-40	KQIGTCGLPGTKCCK	MERCI	POSITIVE	1
5	26-41	QIGTCGLPGTKCCKK	MERCI	POSITIVE	1
6	38-53	CKKPEAAAKVQMTYN	MERCI	POSITIVE	1
7	379-394	GRIGGGGSLSSDQVA	MERCI	POSITIVE	1
8	380-395	RIGGGGSLSSDQVAE	MERCI	POSITIVE	1
9	381-396	IGGGGSLSSDQVAEL	MERCI	POSITIVE	1
10	382-397	GGGGSLSSDQVAELA	MERCI	POSITIVE	1
11	383-398	GGGSLSSDQVAELAA	MERCI	POSITIVE	1
12	482-496	GGGGSNNGNVCLVSD	MERCI	POSITIVE	1
13	483-497	GGGSNNGNVCLVSDA	MERCI	POSITIVE	1
14	484-498	GGSNNGNVCLVSDAK	MERCI	POSITIVE	1
15	485-499	GSNNGNVCLVSDAKM	MERCI	POSITIVE	1
16	486-500	SNNGNVCLVSDAKML	MERCI	POSITIVE	1
17	487-501	NNGNVCLVSDAKMLS	MERCI	POSITIVE	1
18	488-502	NGNVCLVSDAKMLSG	MERCI	POSITIVE	1
19	489-503	GNVCLVSDAKMLSGG	MERCI	POSITIVE	1
20	506-520	SNGNVCLVSDAKMLS	MERCI	POSITIVE	1
21	507-521	NGNVCLVSDAKMLSY	MERCI	POSITIVE	1
22	508-522	GNVCLVSDAKMLSYG	MERCI	POSITIVE	1
23	522-536	GGGGSGNVCLVSDAK	MERCI	POSITIVE	1
24	523-537	GGGSGNVCLVSDAKM	MERCI	POSITIVE	1

#	Start-END	Sequence	Method	Result	Score
25	524-538	GGSGNVCLVSDAKML	MERCI	POSITIVE	1
26	525-539	GSGNVCLVSDAKMLS	MERCI	POSITIVE	1
27	526-540	SGNVCLVSDAKMLSY	MERCI	POSITIVE	1
28	527-541	GNVCLVSDAKMLSYA	MERCI	POSITIVE	1
29	559-573	YAPGGGGSVCLVSDA	MERCI	POSITIVE	2
30	560-574	APGGGGSVCLVSDAK	MERCI	POSITIVE	2
31	561-575	PGGGGSVCLVSDAKM	MERCI	POSITIVE	2
32	562-576	GGGGSVCLVSDAKML	MERCI	POSITIVE	2
33	563-577	GGGSVCLVSDAKMLS	MERCI	POSITIVE	2
34	564-578	GGSVCLVSDAKMLSY	MERCI	POSITIVE	2
35	565-579	GSVCLVSDAKMLSYA	MERCI	POSITIVE	1
36	592-606	HLLAEFFSFFGGGGS	MERCI	POSITIVE	1
37	593-607	LLAEFFSFFGGGGSI	MERCI	POSITIVE	1
38	771-785	IFYASLTYLRRGGGG	MERCI	POSITIVE	7
39	772-786	FYASLTYLRRGGGGS	MERCI	POSITIVE	7
40	773-787	YASLTYLRRGGGGSF	MERCI	POSITIVE	7
41	774-788	ASLTYLRRGGGGSFV	MERCI	POSITIVE	7
42	775-789	SLTYLRRGGGGSFVI	MERCI	POSITIVE	7
43	840-854	STRGRKCCRRKKHHH	MERCI	POSITIVE	1

S12 Table. B Cell discontineous epitopes of CTL & HTL MEVs. Discontinuous B Cell epitopes predicted by ElliPro (IEDB) from CTL & HTL MEVs.

CTL MEV Discontinuous epitopes residues			
#	Residues	Number of residues	Score
1	G1, I2, G3, D4, P5, V6, T7, C8, L9, K10, S11, G12, A13, I14, C15, H16, P17, V18, F19, C20, P21, R22, R23, Y24, K25, Q26, I27, G28, T29, C30, G31, L32, P33, G34, T35, K36, C37, C38, K39, K40, P41, E42, A43, I94, Q95, V545, L546, S547, C548, L549, P550, K551, E552, S559, R561, G562, R563, R568, K569	59	0.747
2	G246, Y288, G289, G290, G291, G292, S293, M294, P295, S296, D297, D298, F299, S300, N301, T302, F303, G304, G305, G306, G307, S308, G321, S322, I323, M324, K325, K326, S327, K329, Y331, G332, G333, N357, I359, Y360, G361, G362, G363, G364, S365, A366, E367, Y389, G390, G391, G392, G393, S394, I395, A396, T397, V398, G407, S408, L409, E410, T411, D412, D413, Y414, N415, G416, I417, Y418, G419, G420, G421, G422, S423, E424, T425, D426, D427, Y428, N429, G430, I431, Y432, G433, G434, G435, G436, S437, S438, Q439, N440, L441, L442, G448, G449, G450, S451, T452, S453, D454, L455, D456, V458, F460, Y461, G462, G463, G464, G465, S466, F467, P468, I469, S470, R471, F473, N474, M475, Y476, G477, G478, G479, G480, S481, R482, G508, G509, S510, K511, Y512, Q514, D516, Q517, F520	130	0.695
3	F56, G57, G58, G59, G60, S61, A62, Q63, A96, G102, G103, G104, G105, G116, G117, G118, G119, S120, L121, A122, M123, D124, E125, G126, Y127, G132, G133, G134, S135, T136, V137, Y138, H139, S141, A142, V143, Y144, G145, G146, G147, G148, S149, A150, V151, Y152, N153, N154, E155, F156, Y157, Y158, G159, G160, G161, G162, S163, A164, E165, N166, G173, G174, G175, G176, S177, N178, Y179, M180, Y181, F187, G188, T198, M199, L200, E201, F202, G203, G204, G205, G206, S207, T208, P209, F210, V211, D212, S213, R214, A215, Y216, G217, G218, G219, G220, S221, G231, G232, G233, G234, S235, T255, D256, V257, V258, Y259, G260, G261, G262, G263, S264, L265, V266, S267, D268, A269, K270, M271, L272, S273, G275, G276, G277, G278, S279, V280, S281, D282, A283, K284, M285, L286, S287	131	0.682
HTL MEV Discontinuous epitopes residues			
#	Residues	Number of residues	Score
1	G1, I2, G3, D4, P5, V6, T7, C8, L9, K10, S11, G12, A13, I14, C15, H16, P17, V18, F19, C20, P21, Y24, K25, Q26, I27, G28, T29, C30, G31, L32, P33, G34, T35, K36, C37, C38, K39, K40, P41, E42, A43, A44, A45, K46, V47, Q48, M49, T50, Y51, N52, W53, T54, Q55, L57, Q58, Y61, G83, G84, G85, S86, L87, A88, L89, S90, K91, Y92, S113, V118, F119, G120, P121, G122, G123, G124, G125, S126, L127, S128, K129, Y130, L131, S132, D133, L134, L135, N160, L161, G162, G163, G164, G165, S166, K167, Y168, L169, F195, G196, P197, N198, L199, Q200, D201, G202, G203, G204, G205, S206, A207, S208, F209, S210, F235, G236, D237, G262, G263, G264, G265, S266, N267, D268, G283, G284, G285, S286, D287, M499, L500, S501, G502, G503, G504, G505	133	0.745
2	S71, G319, V320, F321, G322, G323, G324, G325, R331, N333, W334, I335, S336, A337, G338, V339, F340, L341, G342, G343, G344, G345, S346, I347, P348, R349, E350, F351, M352, I353, Y354, D355, D356, V357, F358, I359, D360, N361, G362, G363, G364, G365, S366, F367, M368, I369, Y370, D371, D372, V373, F374, I375, D376, N377, T378, G379, R380, I381, G382, G383, G384, G385, S386, L387, S388, S389, D390, Q391, V392, A393, E394, L395, Q400, E401, G402, G403, G404, G405, S406, S407, S408, L433, A434, A435, A436, V437, Q438, E439, T440, G442, G443, G444, G445, S446, D447, Q448, A450, E451, A454, Q457, E458, T459, S460, A461, G462, G463, G464, G465, S466, Q467, V468, A469, E470, L471, A472, A473, A474, V475, Q476, E477, T478, S479, A480, G481, G482, G483, G484, G485, S486, N487, N488, G489, N490, S514, D515, A516, K517, M518, G522, G523, G524, G525, S526, G527, D553, A554, K555, M556, S558, Y559, A560, P561, G562, G563, G564, G565, A573, K574, M575, L576, S577, Y578, A579, P580, E581, G582, G583, G584, G585, S586, N587, D589, F601, G602, G603, G604, G605, S606, I607, D608, N609, F616, R640, N647, I648, H649, L650, L651, A652, E653, F654, F655, S656, F657, F658, R659, T660, F661, G662, G663, G664, G665, S666, I667, H668, L669, L670, A671, F673, T679, F680, G681, G682, G683, G684, G702, G704, G705, S706, E707, L708, A709, S710, F711, L712, M713, D714, R715, R716, V717, I718, R721, G722, G723, G724, G725, S726, L727, A728, S729, F730, L731, D733, P739, A741, G742, G743, G744, G745, S786, F787, V788, I789, F790, A792, S793, L794, T795, Y796, L797, R798, R799, G800, I801, E802, A803, A804, A805, K806, G807, I808, I809, N810, T811, L812, Q813, K814, Y815, Y816, R820, G821, G822, A825, V826, L827, S828, C829, L830, P831, K832, E833, E834, Q835, I836, G837, K838, T841, R842, G843, K845, C846, C847, R848, R849, K850, K851, H852, H853, H854, H855, H856	311	0.687
3	D273, D292, I294, N295, I297, S298, A299, G300, V301, G302, G303	11	0.624
4	G105, S106, A107, L108, S109, K110, Y111, E420, T421, G422, G423, G424, G425, S426, S427, D428	16	0.598
5	G139, P140, N141, G142, G143, G144, G145, S146, S147	9	0.558

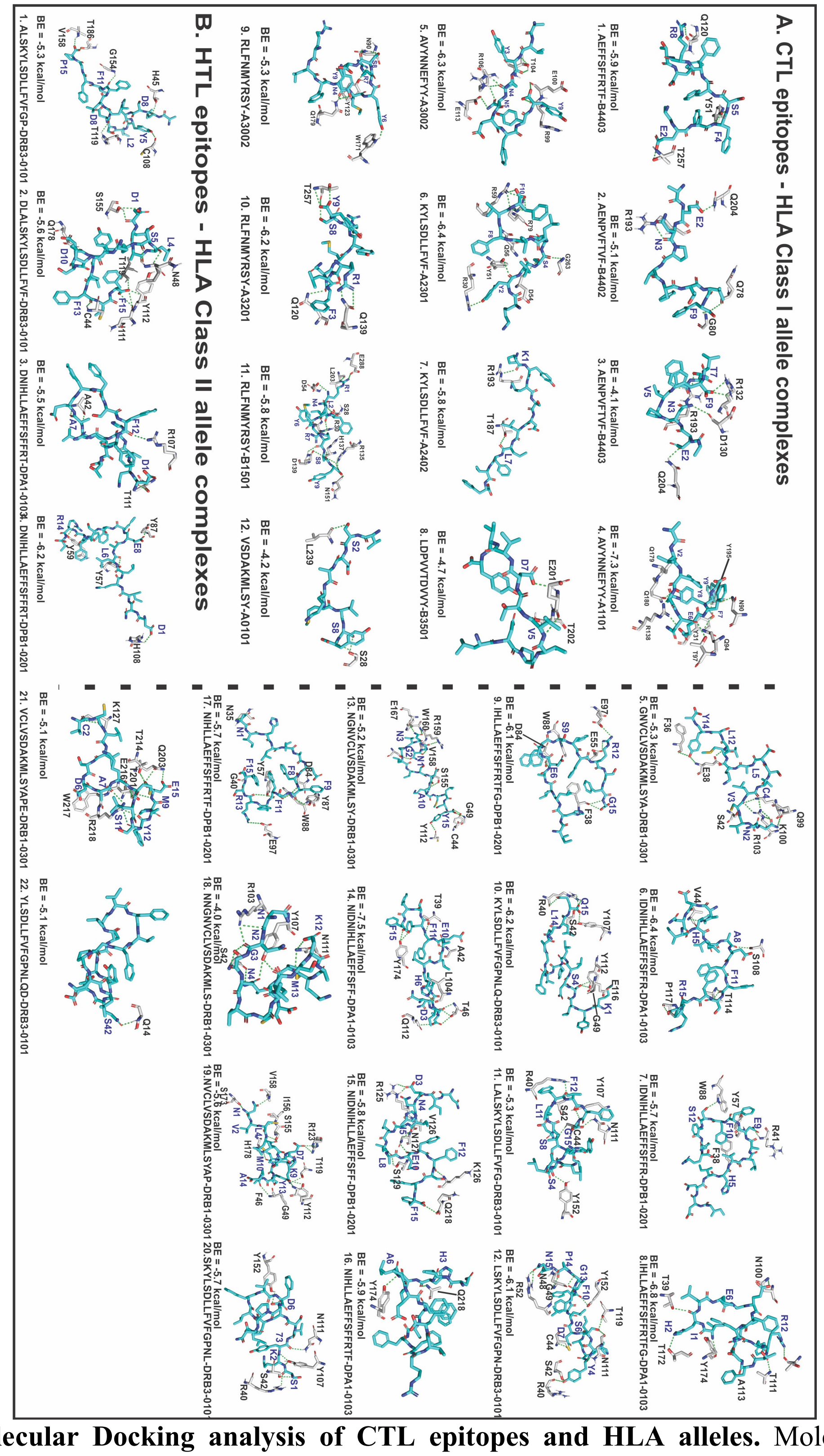

S1_Fig. (A) Molecular Docking analysis of CTL epitopes and HLA alleles. Molecular docking of selected CTL epitopes (cyan sticks) with their respective HLA class I allele binders (gray sticks). The study shows the docked complexes to have significantly negative binding energy along with hydrogen bonds (green dots) formation in the complex interface. **(B) Molecular Docking analysis of HTL epitopes and HLA alleles.** Molecular docking of selected HTL epitopes (cyan sticks) with their respective HLA class II allele binders (gray sticks). The study shows the docked complexes to have significantly negative binding energy along with hydrogen bonds (green dots) formation in the complex interface. (*) Indicates binding energy, shown in kcal/mol.

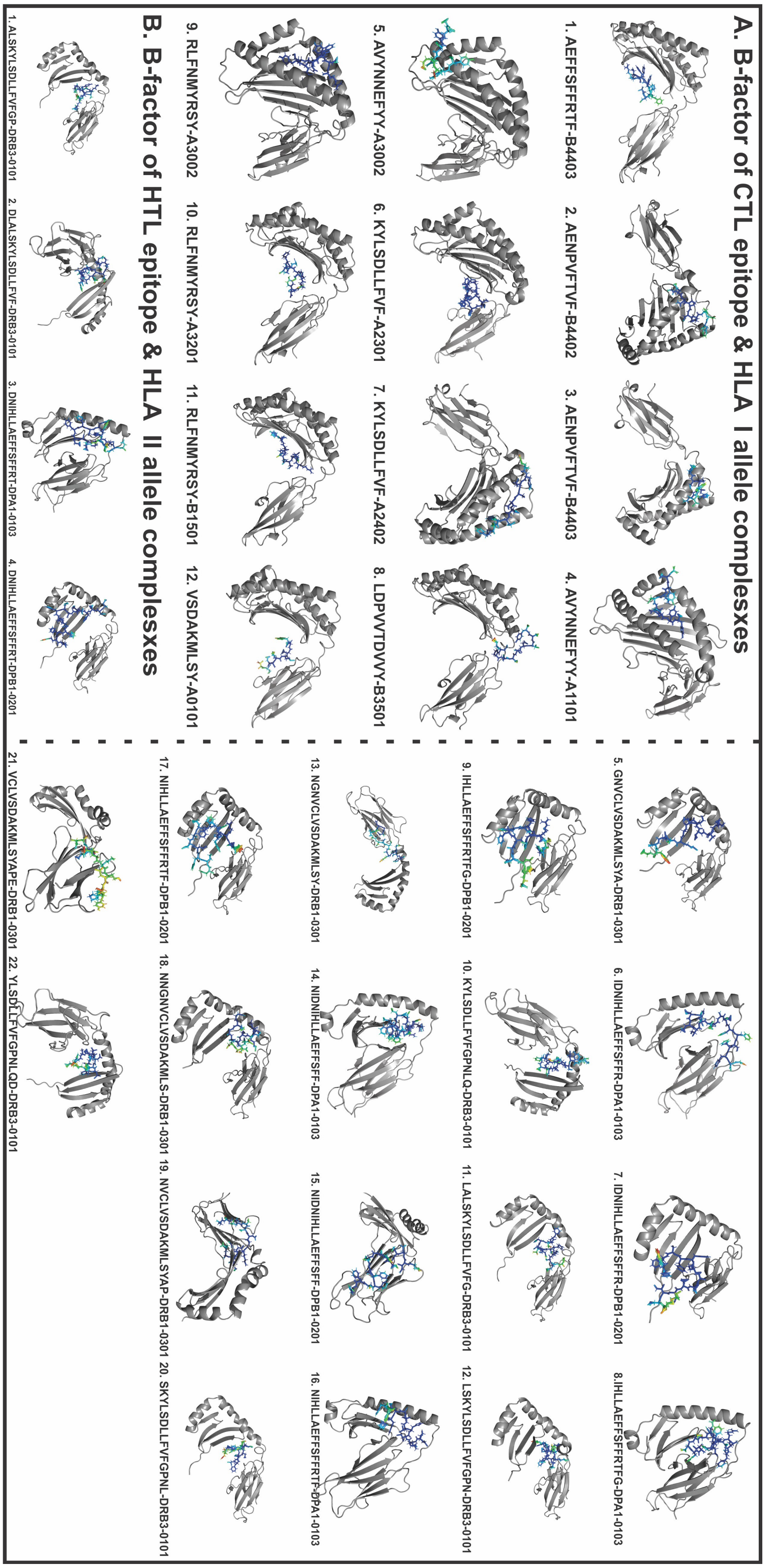

S2_Fig. (A) B-Factor of CTL epitope – HLA class I allele complexes **(B)** B-Factor of HTL epitope – HLA class II allele complexes. Epitopes are shown in sticks and HLA alleles are shown in gray cartoons. B-factor is indicated by rainbow (VIBGYOR) colour, blue for stable region and red for most unstable region.

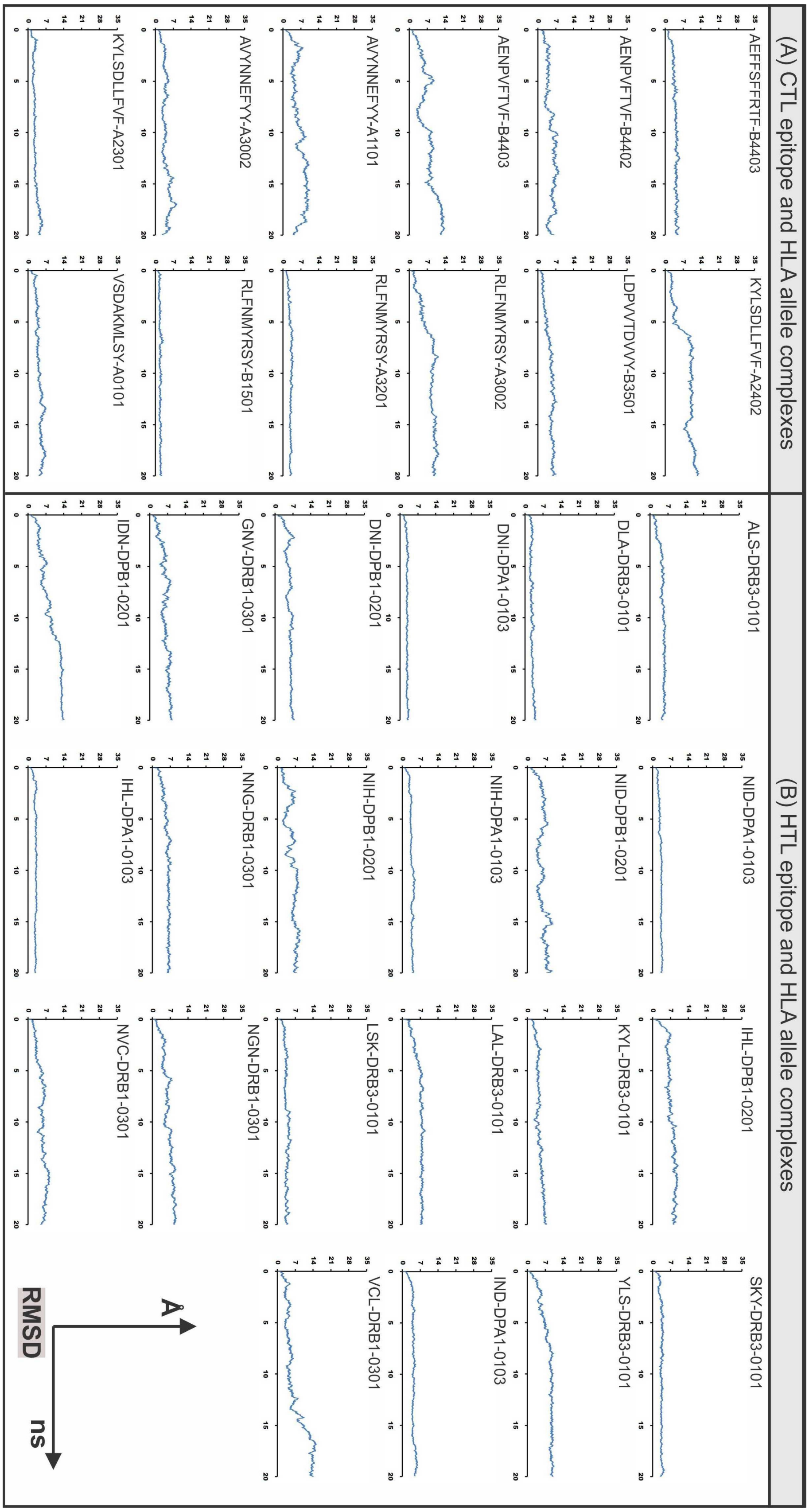

S3_Fig. (A) Molecular Dynamics simulation analysis of CTL epitopes and HLA allele complexes. Molecular Dynamics simulation study reveals a stable nature of the CTL-HLA allele complexes achieving a convergence within 20 ns time window. **(B) Molecular Dynamics simulation analysis of CTL epitopes and HLA allele complexes.** Molecular Dynamics simulation study reveals a stable nature of the HTL-HLA allele complexes achieving a convergence within 20 ns time window.

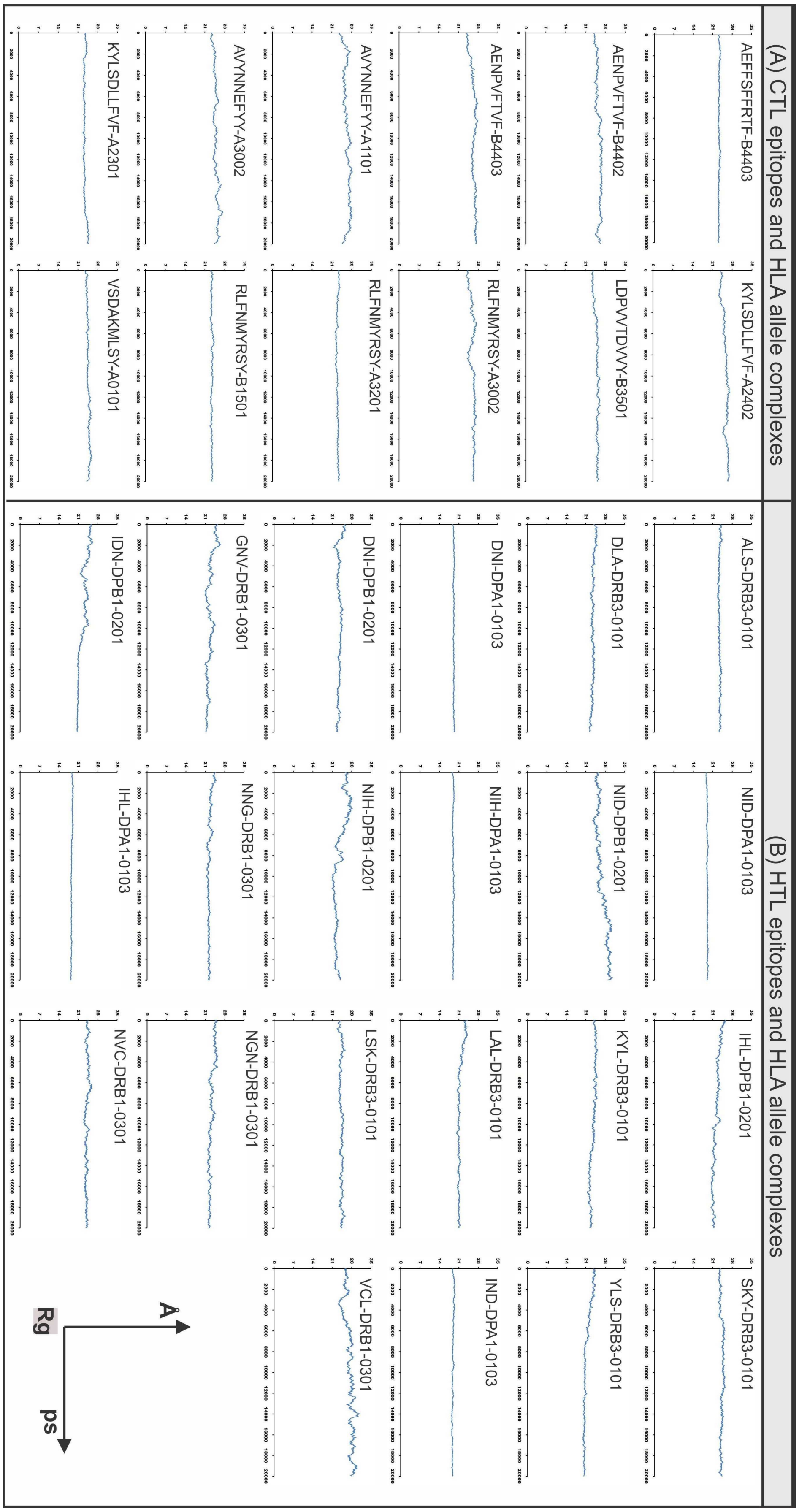

S4_Fig. (A) Rg (radius of gyration) for the CTL epitope – HLA class I allele complexes, across the time window of 20 nano second. **(B)** Rg for the HTL epitope – HLA class II allele complexes, across the time window of 20 nano second.

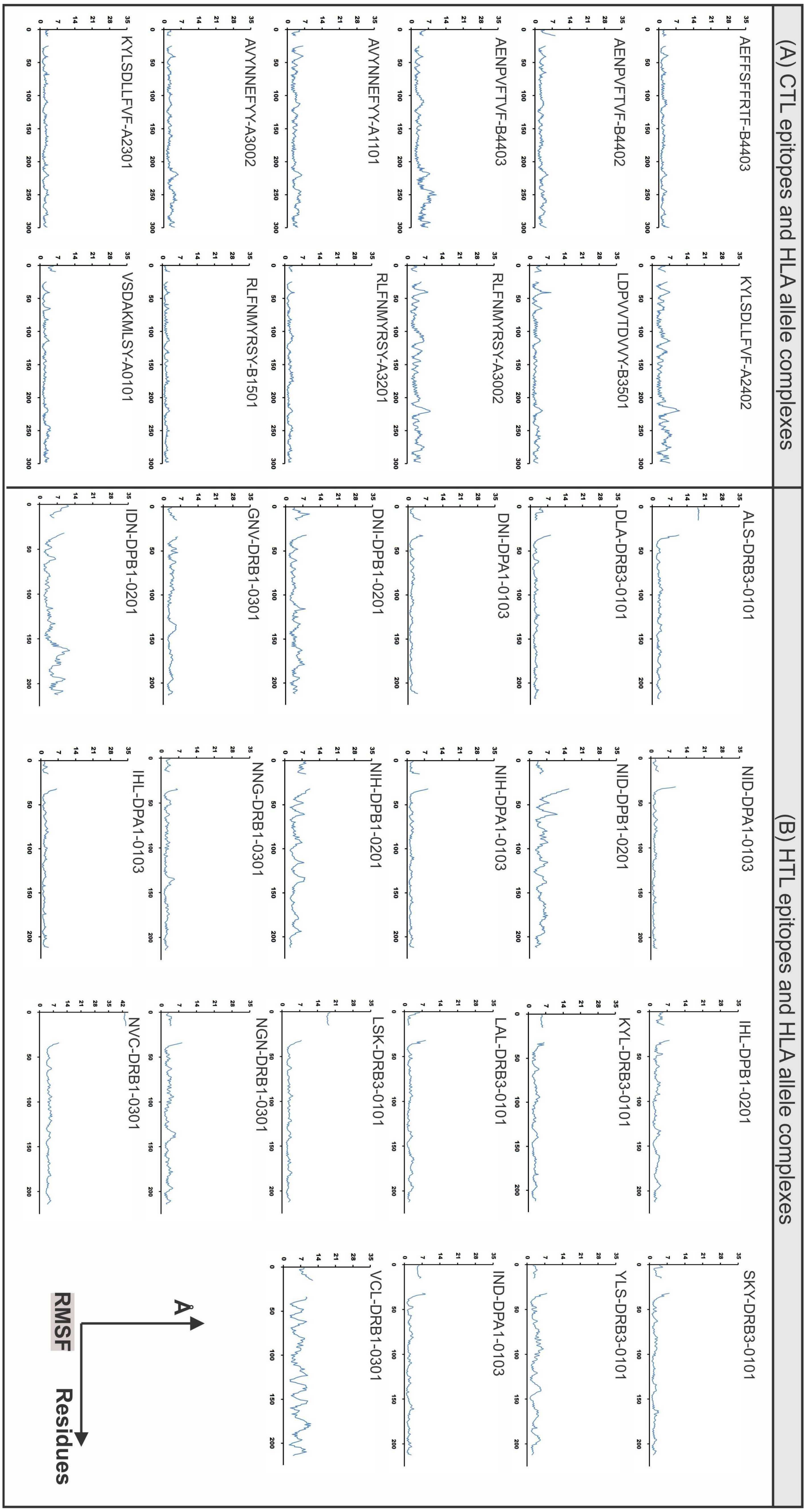

S5_Fig. (A) RMS fluctuation in angstrom for all the amino acid residues of the CTL epitope – HLA class I allele complexes. **(B)** RMS fluctuation in angstrom for all the amino acid residues of the HTL epitope – HLA class II allele complexes.

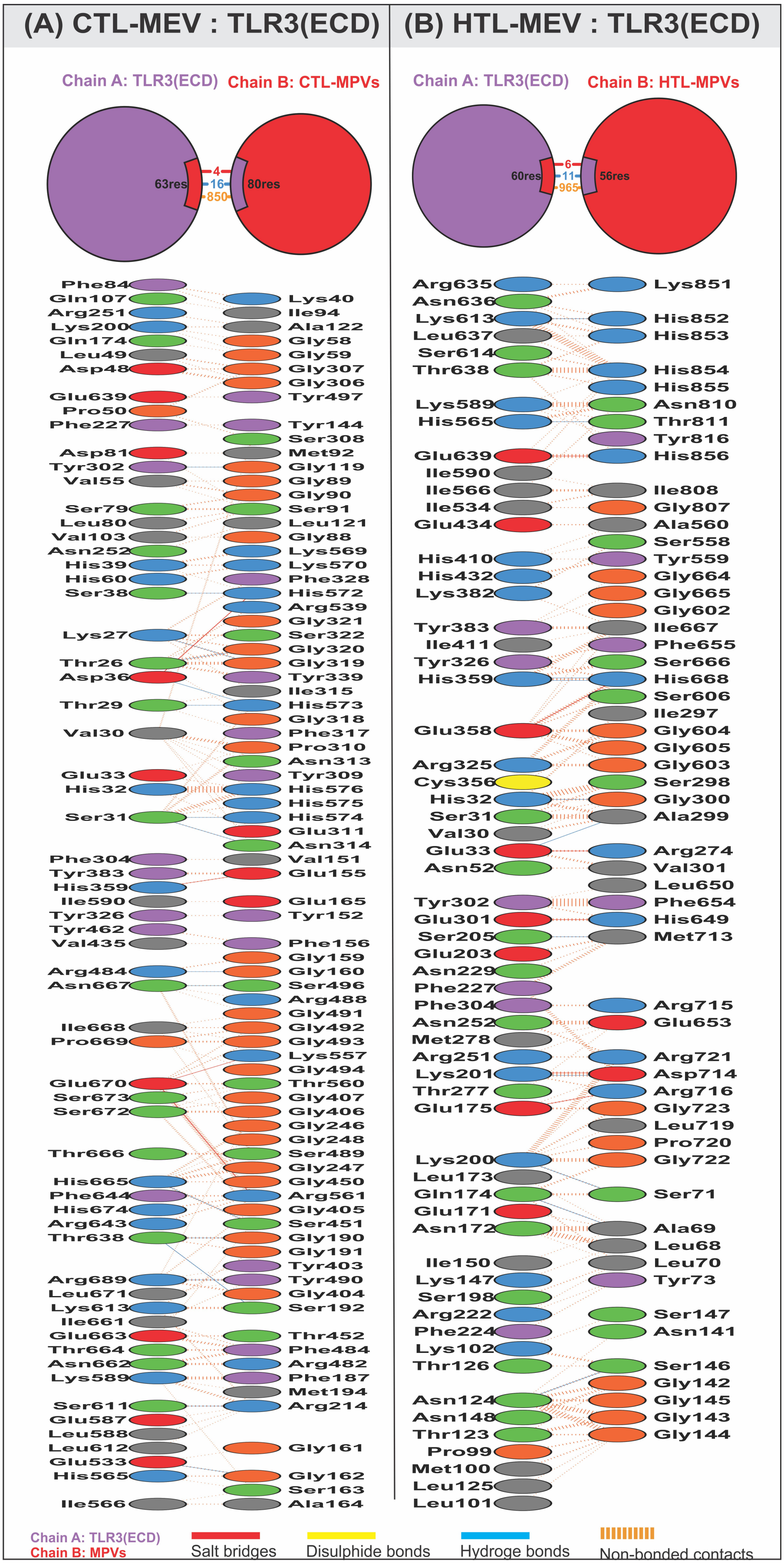

S6_Fig. (A) & (B) Show interaction of CTL and HTL MEVs with TLR3(ECD). The residues involved in interaction and forming different bonds are shown. Notably, CTL MEV complex with TLR3(ECD) forms 16 hydrogen bonds whereas HTL MEV complex with TLR3(ECD) forms 11 hydrogen bonds.
